# Global trends and epidemiological impact of metabolic risk factors on atrial fibrillation and atrial flutter from 1990 to 2021

**DOI:** 10.1038/s41598-025-88744-4

**Published:** 2025-02-07

**Authors:** Junqing Liang, Jun Shen, Yankai Guo, Manzeremu Rejiepu, Xiuwen Ling, Xiaoyan Wang, Yi Jian, Xing Zhang, Shijie Shao, Baopeng Tang, Ling Zhang

**Affiliations:** 1 Xinjiang Key Laboratory of Cardiac Electrophysiology and Cardiac Remodeling, The First Affiliated Hospital of Xinjiang Medical University, Urumqi, 83000 Xinjiang People’s Republic of China; 2 Cardiac Pacing and Electrophysiology Department, The First Affiliated Hospital of Xinjiang Medical University, Urumqi, Xinjiang People’s Republic of China; 3 Emergency Trauma Center, The First Affiliated Hospital of Xinjiang Medical University, Urumqi, Xinjiang People’s Republic of China; 4 Department of Cardiology, Renmin Hospital, Hubei University of Medicine, Shiyan, People’s Republic of China

**Keywords:** Global burden of disease, Atrial fibrillation/atrial flutter, Metabolic risk factors

## Abstract

Atrial fibrillation (AF) and atrial flutter (AFL) are cardiac arrhythmias associated with high morbidity and mortality. This cross-sectional observational study assesses the epidemiological burden of AF/AFL concerning metabolic risk factors using the Global Burden of Disease (GBD) 2021 database. Age-standardized disability-adjusted life years rate (ASDR), mortality, and estimated annual percentage change were calculated. The Bayesian Age-Period-Cohort model was utilized to predict future trends in ASDR and age-standardized mortality rate (ASMR) for AF/AFL 2030. Between 1990 and 2021, ASDR rose from 34.22 to 34.94, whereas ASMR increased from 1.46 to 1.50 per 100,000 people. High/high–middle Socio-Demographic Index (SDI) regions showed decreasing trends in ASDR and ASMR, whereas low and middle SDI regions showed increasing trends. In 2021, the highest ASDR and ASMR were recorded in Australasia, North America, and Western Europe, with significant country-level variability. Projections indicate a declining trend in ASMR and stable ASDR through 2030. The global rise in AF/AFL attributable to metabolic risk factors necessitates comprehensive and region-specific public health strategies. Further research is warranted to develop and implement effective measures to mitigate these conditions and improve cardiovascular health outcomes worldwide.

**Supplementary Information:**

The online version contains supplementary material available at 10.1038/s41598-025-88744-4.

## Introduction

Atrial fibrillation and atrial flutter (AF/AFL) are the most common forms of arrhythmia. These arrhythmias contribute to a notable incidence of heart failure and ischemic stroke, ultimately resulting in increased cardiovascular and cerebrovascular morbidity and mortality. The resulting adverse events lead to considerable healthcare costs, placing a substantial burden on public health^1,2^. Global economic expansion has substantially heightened the prevalence of metabolic risk factors^3^, including high fasting plasma glucose, high low-density lipoprotein cholesterol, high systolic blood pressure (SBP), high body mass index (BMI), low bone mineral density, and kidney dysfunction^4^. These trends underscore the need for comprehensive public health strategies and measures to mitigate their effects on population health^5^.

An increasing body of research suggests a strong correlation between metabolic risk factors and AF/AFL^6^. These risk factors not only accelerate the incidence of AF/AFL but also intensify their progression by mediating pathophysiological mechanisms via various processes^7^. Addressing these metabolic disorders is essential to lowering the prevalence of AF/AFL, improving heart health, and reducing associated complications. Nonetheless, extensive epidemiological information on the impact of AF and AFL on metabolic risk factors in 2021 is lacking. The knowledge gap warrants additional studies to deepen the comprehension of epidemiology in this context and to formulate specific interventions.

The Global Burden of Disease (GBD) database provides comprehensive and comparable data on global diseases, injuries, and risk factors, acting as a global center for health research^8^. The database includes the incidence and prevalence of numerous diseases and injuries worldwide, as well as their classification by region, age, sex, and time frame.

The focus of this study was on metabolic risk factors, especially high systolic blood pressure (SBP) and high body mass index (BMI), as classified by the official language of the GBD 2021 database^9^. According to this database, elevated SBP is classified as SBP levels surpassing the theoretically minimal risk exposure limit, which is 105–115 mmHg specifically^9^. BMI is calculated as the squared proportion of weight (in kilograms) to height (in meters). The GBD 2021 database defines high BMI in adults (≥ 20 years) as exceeding the theoretical minimum risk exposure level of 20–25 kg/m^2^, with overweight (BMI ≥ 25 kg/m^2^) and obesity (BMI ≥ 30 kg/m^2^) categorized according to global World Health Organization standards. For children and adolescents (2–19 years), the determination of high BMI is based on age- and sex-specific thresholds as stipulated by the International Obesity Task Force standards^9^.

This study utilized the GBD 2021 database to download information related to metabolic risk factors associated with AF/AFL. An extensive analysis was carried out to evaluate the epidemiological impact of these conditions at various stages, encompassing factors, such as region, country, time, sex, and the Socio-Demographic Index (SDI). This study intends to offer conceptual directions for creating strategies to prevent AF/AFL associated with metabolic risk factors worldwide.

## Methods

### Data sources

Epidemiological information on AF/AFL associated with metabolic risk factors from 1990 to 2021 was obtained from the Global Health Data Exchange query tool (https://vizhub.healthdata.org/gbd-results), which is a digital repository of the GBD 2021 database^10^. The database includes evaluations of 371 causes of death or injuries, in addition to 88 risk factors, across 21 regions and 204 countries. Based on the SDI, these 204 nations are divided into five distinct zones: low SDI, low-middle SDI, middle SDI, middle-high SDI, and high SDI^11^.

### Statistical analysis

In this study, disability-adjusted life years (DALYs), mortality, and estimated annual percentage change (EAPC) were calculated to evaluate the worldwide impact of AF/AFL associated with metabolic risk factors. DALYs reflect the cumulative effect of illness and injury on health and are determined as the sum of years of life lost (YLLs) because of premature death and the years lived with disability (YLDs), denoted as DALYs = YLLs + YLDs^12^. YLLs are calculated as the sum of differences between the actual age at death and the expected lifespan of each deceased individual^13^. YLDs are calculated by multiplying the prevalence of an illness or injury by its mean duration and the disability severity weights^14^. EAPC was used to measure the annual changes in the disease burden over time, enabling the evaluation of health patterns and the effectiveness of public health strategies^15^. EAPC is calculated through regression analyses to determine the rate of change in DALYs annually.

The Bayesian Age-Period-Cohort (BAPC) model represents an advanced forecasting model that merges Bayesian statistical techniques with age-period-cohort analysis to investigate the temporal patterns of disease prevalence^16^. By separating the impacts of age, period, and cohort^17^, the BAPC model facilitates accurate forecasts that are vital for public health policy professionals in formulating and executing more effective strategies. Our current research utilized the BAPC model to forecast the ASDR and ASMR for AF/AFL associated with metabolic risk factors through 2030.

All statistical analyses were conducted using R (version 4.3.2).A *p*-value < 0.05 indicated statistical significance.

## Results

### Trends in the global burden of AF/AFL related to metabolic risk factors

Globally, the incidence of AF/AFL associated with metabolic risk factors showed a consistent upward trajectory from 1990 to 2021. The ASDR caused by metabolic risk factors per 100,000 people increased from 34.22 (95% UI 14.16–53.90) in 1990 to 34.94 (95% UI 15.64–54.66) in 2021. This value corresponds to an EAPC of 0.48 (95% CI − 1.16–2.15) per 100,000 people. Similarly, the ASMR increased from 1.46 (95% UI 0.61–2.25) in 1990 to 1.50 (95% UI 0.68–2.33) per 100,000 people in 2021. This value corresponds to an EAPC of 0.53 (95% CI − 1.18–2.27) per 100,000 people (Table 1). Overall, these results show a significant increase in the worldwide incidence of AF/AFL associated with metabolic risk factors from 1990 to 2021.

**Table 1:** ASDR and ASMR of AF/AFL associated with metabolic risk factors between 1990 and 2021, along with their temporal trends.

Characteristics	1990		2021		EAPCs(1990–2021)	
	ASDR (95% UI)	ASMR(95% UI)	ASDR(95% UI)	ASMR(95% UI)	ASDR(95% CI)	ASMR (95% CI)
Global	34.22(14.16–53.90)	1.46(0.61–2.25)	34.94(15.64–54.66)	1.50(0.68–2.33)	0.48(− 1.16–2.15)	0.53(− 1.18–2.27)
Sex						
Males	35.51(14.05–56.86)	1.35(0.54–2.14)	36.91(16.03–58.83)	1.44(0.64–2.25)	0.78(− 1.04–2.64)	0.92(− 1.02–2.89)
Females	32.78(14.05–50.65)	1.51(0.64–2.31)	33.00(15.23–50.93)	1.53(0.70–2.39)	0.31(− 1.22–1.86)	0.40(− 1.21–2.04)
Sociodemographic index						
Low	20.60(7.18–35.76)	0.84(0.25–1.57)	25.15(9.19–42.64)	1.10(0.39–1.94)	2.02(0.14–3.93)	2.38(0.39–4.41)
Low-middle	23.23(8.65–38.93)	0.89(0.32–1.51)	30.94(12.57–48.84)	1.33(0.54–2.10)	1.98(0.55–3.42)	2.31(0.86–3.78)
Middle	26.39(9.94–43.34)	1.18(0.46–1.93)	32.45(13.5–52.12)	1.42(0.60–2.24)	1.63(0.36–2.92)	1.61(0.25–2.99)
High-middle	34.66(14.55–53.56)	1.50(0.65–2.30)	34.51(15.78–53.84)	1.52(0.70–2.35)	0.38(− 0.51–1.28)	0.55(− 0.39–1.51)
High	44.86(19.32–69.59)	1.79(0.78–2.75)	41.67(19.79–64.99)	1.65(0.77–2.54)	0.122(− 0.65–0.90)	0.20(− 0.64–1.06)
Region						
Andean Latin America	21.41(8.45–36.55)	0.89(0.35–1.54)	32.21(14.38–51.31)	1.26(0.57–2.00)	2.01(0.68–3.36)	2.01(0.36–3.68)
Australasia	57.27(24.26–88.45)	2.60(1.10–4.01)	52.62(24.91–81.13)	2.28(1.10–3.50)	0.36(− 0.83–1.56)	0.31(− 0.96–1.61)
Caribbean	35.77(14.10–57.76)	1.60(0.64–2.54)	38.15(17.22–60.08)	1.56(0.71–2.44)	0.88(− 0.70–2.48)	0.71(− 1.05–2.51)
Central Asia	24.21(10.43–38.58)	0.68(0.31–1.04)	29.75(13.61–45.78)	0.95(0.45–1.40)	0.82(− 0.44–2.10)	1.15(− 0.16–2.47)
Central Europe	46.94(21.65–70.22)	2.05(0.94–3.01)	44.45(21.17–66.52)	1.77(0.88–2.62)	0.08(− 1.12–1.30)	− 0.01(− 1.25–1.26)
Central Latin America	36.26(15.47–57.11)	1.52(0.66–2.38)	42.81(19.81–65.43)	1.70(0.82–2.52)	1.05(− 0.24–2.36)	0.97(− 0.48–2.43)
Central Sub-Saharan Africa	30.18(10.57–51.38)	1.35(0.42–2.47)	32.53(12.00–54.60)	1.53(0.51–2.82)	1.76(− 1.04–4.63)	2.05(− 0.96–5.15)
East Asia	24.15(8.21–41.95)	1.32(0.45–2.38)	28.56(11.45–47.34)	1.36(0.54–2.27)	2.94(− 0.40–6.34)	2.49(− 1.01–6.20)
Eastern Europe	37.19(16.41–56.71)	1.48(0.68–2.28)	44.36(21.64–66.53)	1.78(0.89–2.57)	0.87(− 0.28–2.03)	0.83(− 0.35–2.02)
Eastern Sub-Saharan Africa	18.34(5.96–32.54)	0.76(0.24–1.49)	23.71(8.40–40.12)	0.95(0.31–1.76)	2.13(− 0.91–5.26)	2.18(− 1.14–5.61)
High-income Asia Pacific	28.48(9.96–46.06)	1.06(0.39–1.69)	19.86(7.40–33.15)	0.72(0.27–1.20)	− 0.12(− 3.36–3.22)	− 0.48(− 3.92–3.09)
High-income North America	44.84(20.08–69.52)	1.49(0.67–2.28)	51.45(26.67–79.09)	1.88(0.95–2.85)	0.71(− 0.26–1.69)	1.03(− 0.13–2.19)
North Africa and Middle East	23.84(10.58–37.35)	1.15(0.49–1.86)	29.03(15.30–43.9)	1.46(0.74–2.17)	1.15(− 0.08–2.39)	1.43(0.13–2.74)
Oceania	24.24(9.72–41.37)	0.98(0.38–1.68)	29.97(13.43–48.01)	1.14(0.48–1.88)	0.97(− 0.51–2.46)	0.72(− 0.93–2.40)
South Asia	19.92(6.87–35.16)	0.66(0.19–1.27)	26.86(9.99–44.94)	1.10(0.39–1.91)	2.99(− 0.48–6.57)	3.85(0.10–7.75)
Southeast Asia	31.65(11.02–51.61)	1.29(0.48–2.13)	39.56(15.39–64.06)	1.79(0.69–2.85)	2.50(− 0.98–6.11)	3.16(− 0.84–7.32)
Southern Latin America	26.44(11.63–41.66)	1.20(0.52–1.88)	29.35(14.02–44.15)	1.41(0.68–2.10)	1.19(− 0.01–2.41)	1.79(0.42–3.17)
Southern Sub-Saharan Africa	27.89(11.64–44.05)	1.02(0.44–1.63)	37.60(18.51–55.91)	1.63(0.79–2.43)	1.47(0.20–2.76)	2.09(0.71–3.49)
Tropical Latin America	39.05(15.85–63.20)	1.55(0.63–2.47)	44.15(20.24–68.97)	1.73(0.79–2.65)	1.05(− 0.39–2.52)	1.25(− 0.36–2.88)
Western Europe	50.95((21.65–79.21))	2.10(0.92–3.20)	47.14(22.27–73.95)	1.98(0.92–3.06)	0.17(− 1.27–1.64)	0.45(− 1.06–1.97)
Western Sub-Saharan Africa	24.20(9.09–40.64)	1.28(0.50–2.17)	31.94(13.29–50.07)	1.64(0.71–2.56)	1.66(− 0.51–3.89)	1.50(− 0.72–3.76)

AF/AFL = atrial fibrillation/atrial flutter; ASDR = age-standardized rates of disability-adjusted life years; ASMR = age-standardized mortality rate; and EAPC = estimated annual percentage change.

### Global burden of metabolic risk factors related to AF/AFL by sex and age

**Fig. 1:**
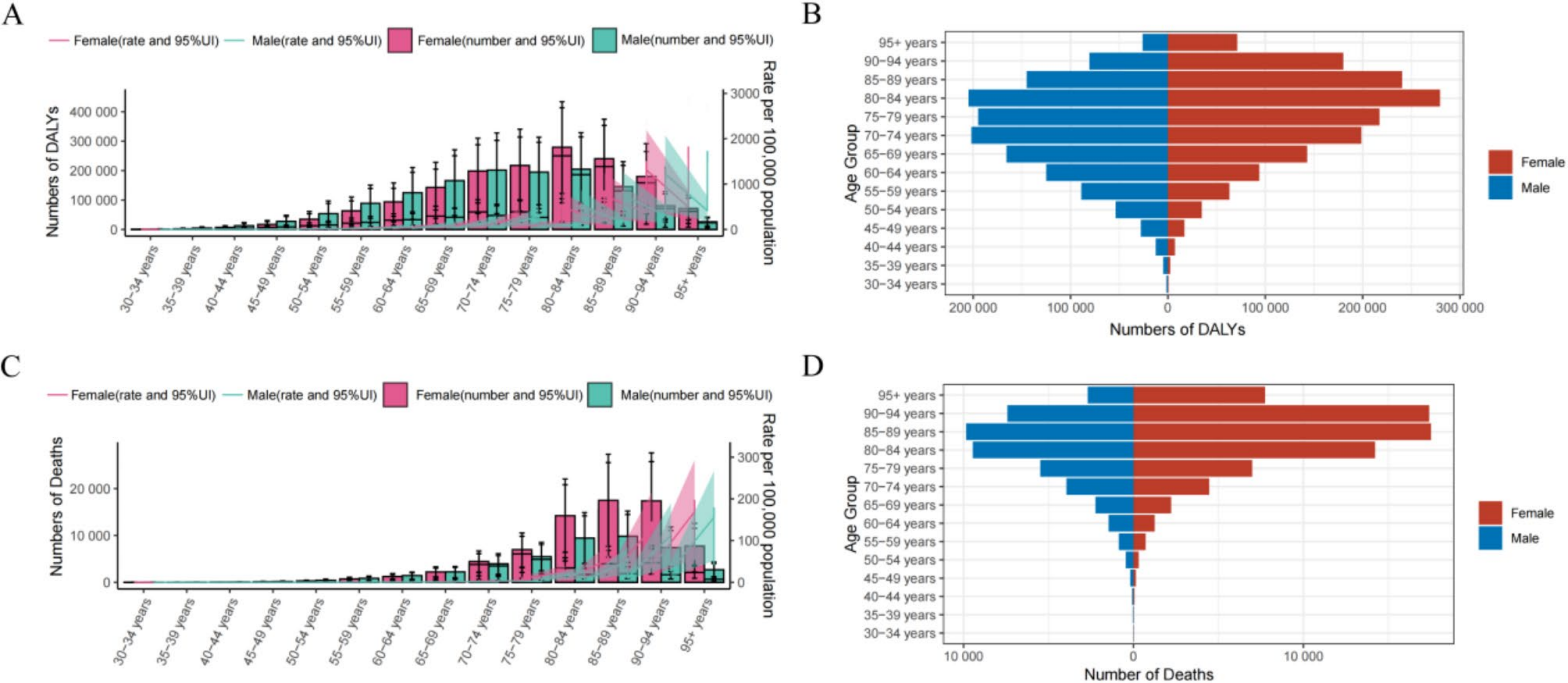
DALYs and deaths attributable to metabolic risk factors by sex and age in 2021 (**A**) DALYs presented as both the absolute number and rate per 100,000 people. (**B**) The number of DALYs. (**C**) Deaths presented as both the absolute number and rate per 100,000 people. (**D**) The number of deaths. DALYs = disability-adjusted life years.

In 2021, AF/AFL associated with metabolic risk factors constituted a major health challenge worldwide, marked by distinct variances across sex and age demographics. The number of DALYs for males was 204,846.91 (95% UI 88,936.52–329,023.77) and for females it was 279,428 (95% UI 121,942.84–434,281.81) (Table S1). The data indicated an initial rise in DALYs with age, followed by a decline at advanced ages (Fig. 1A,B). This trend was observed in both sexes, though females demonstrated a significantly higher disease burden than males.

The trend in the number of deaths mirrored the trends observed for DALYs, with an increase in deaths with age peaking in the 85–89 age group, followed by a decline (Fig. 1 C,D). For males, the number of deaths was 9,826.60 (95% UI 4,232.90–15,212.61) and for females it was 17,503.22 (95% UI 7,672.95–27,335.52) (Table S1). Consistent with the DALYs trends, the mortality burden was significantly higher in females than in males. Thus, AF/AFL induced by metabolic risk factors substantially influences the health of older adults, particularly among older females.

### Trends in the burden of metabolic risk factors for AF/AFL by SDI regions and sex

**Fig. 2:**
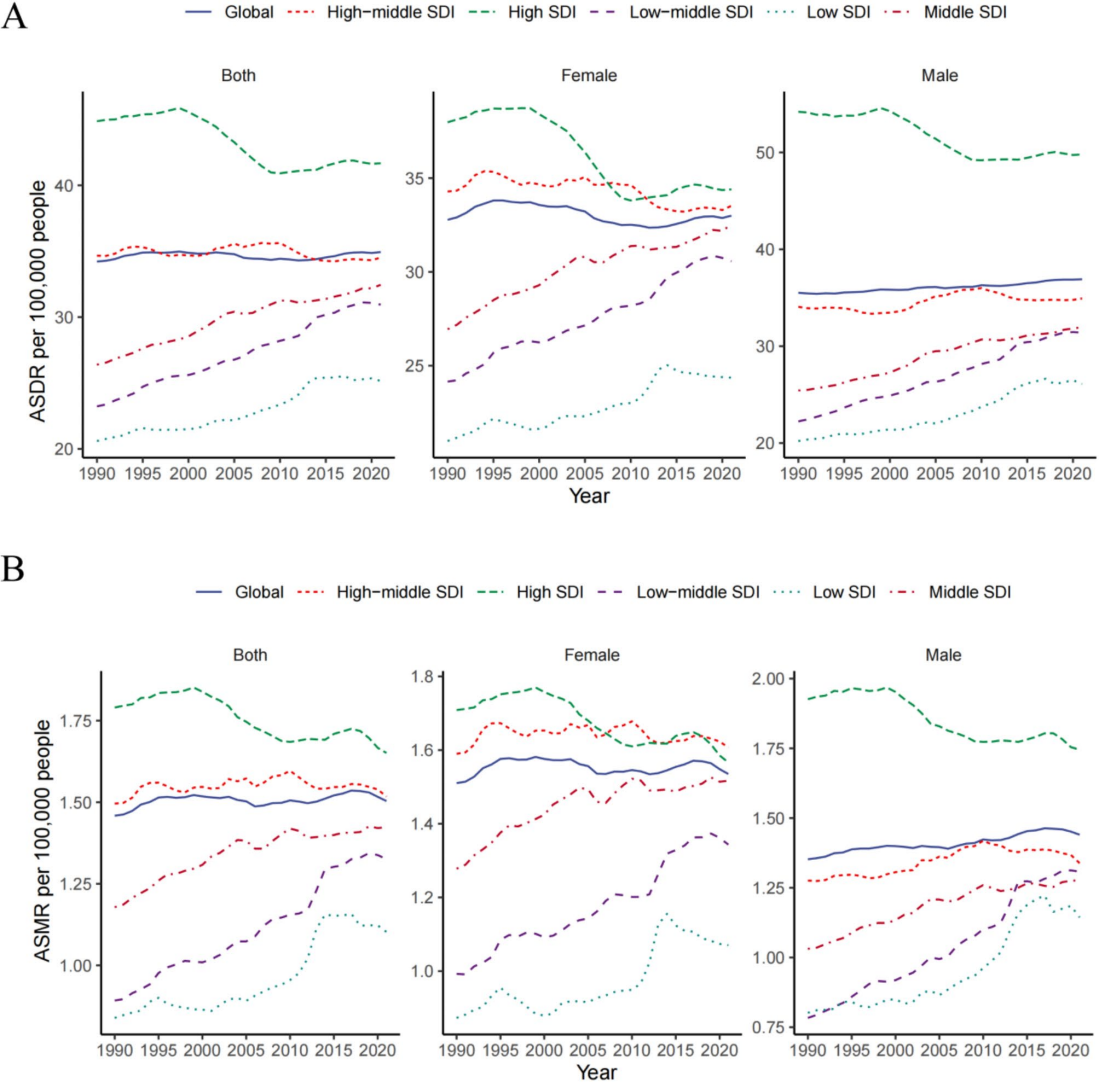
Global and AF/AFL burden associated with metabolic risk factors for five SDI regions (**A**) ASDR per 100,000 people between 1990 and 2021 stratified by sex and SDI. (**B**) ASMR per 100,000 people between 1990 and 2021 stratified by sex and SDI. AF/AFL = atrial fibrillation/atrial flutter; ASDR = age-standardized rates of disability-adjusted life years; ASMR = age-standardized mortality; and SDI = socio-demographic index.

Between 1990 and 2021, the burden of AF/AFL due to metabolic risk factors showed significant geographic variations across different SDI regions. In terms of the ASDR, high-SDI regions showed a consistent annual decline, which was similarly observed in high-middle SDI regions. Conversely, low SDI regions exhibited year-on-year increasing trends, which was consistent for both males and females. Regarding ASMR, high SDI regions demonstrated a fluctuating yet overall decreasing annual trend, similar to high-middle SDI regions (Fig. 2A). In contrast, low, low-middle, and middle SDI regions showed fluctuating but overall increasing year-to-year trends. After stratification by sex, females exhibited a fluctuating annual upward trend across all SDI regions, consistent with the general trend. In contrast, males exhibited an annual decline exclusively in high SDI regions, whereas other SDI regions showed an annual upward trend (Fig. 2B).

### Burden of metabolic risk factors for AF/AFL by GBD regions

In 2021, among the 21 GBD regions, Australasia, high-income North America, and Western Europe demonstrated the highest ASDR per 100,000 people, with values of 52.62 (95% UI 24.91–81.13), 51.45 (95% UI 26.67–79.09), and 47.14 (95% UI 22.27–73.95), respectively (Figure S1A, Table 1). These regions also ranked highly in terms of ASMR per 100,000 people, with values of 2.28 (95% UI 1.10–3.35) for Australasia, 1.98 (95% UI 0.92–3.06) for Western Europe, and 1.88 (95% UI 0.95–2.85) for high-income North America (Figure S1B, Table 1).

**Fig. 3:**
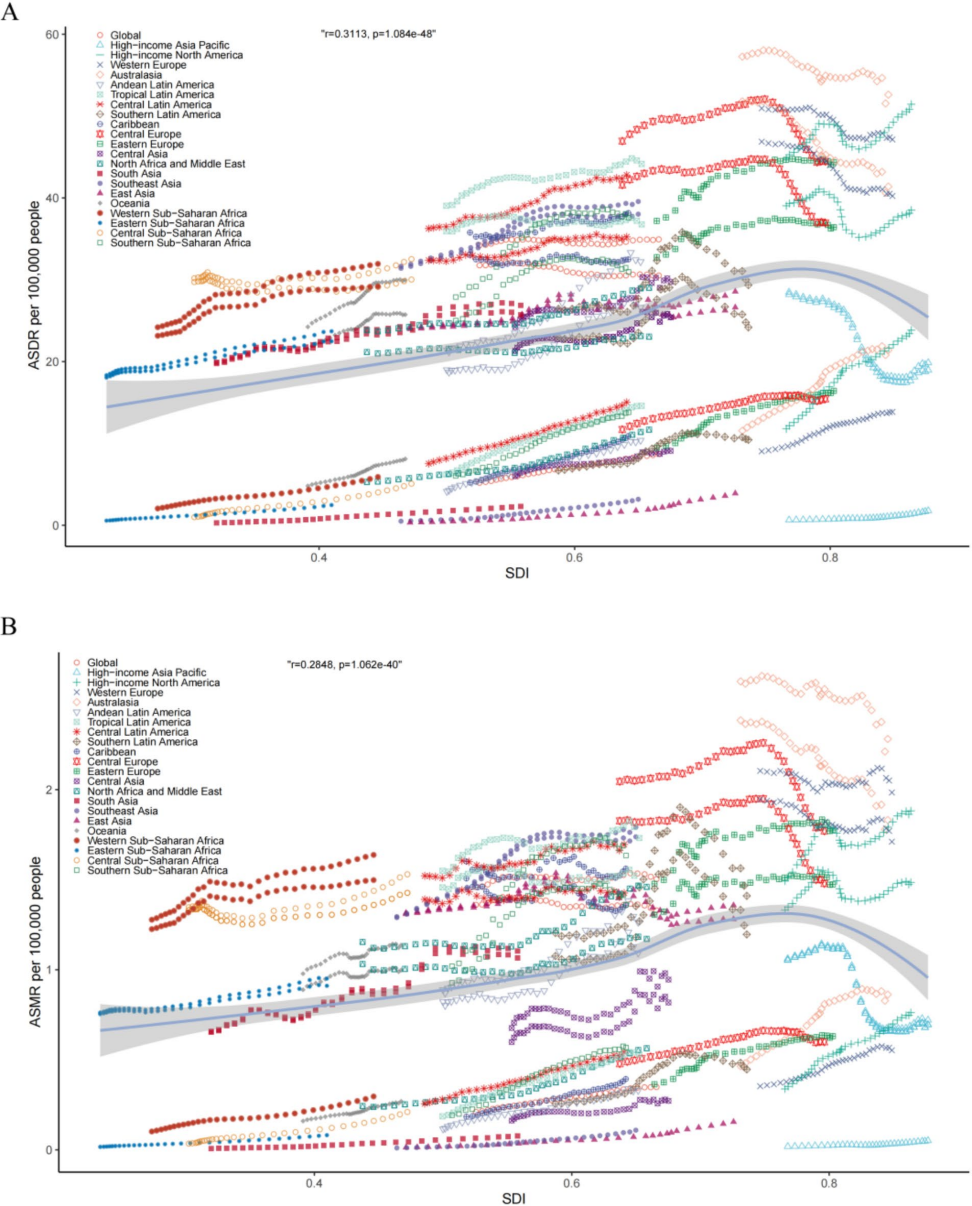
AF/AFL burden associated with metabolic risk factors across 21 GBD regions by SDI between 1990 and 2021 (**A**) ASDR per 100,000 people. (**B**) ASMR per 100,000 people. AF/AFL = atrial fibrillation /atrial flutter; ASDR = age-standardized rates of disability-adjusted life years; ASMR = age-standardized mortality; and SDI = socio-demographic index.

Sex stratification suggested regional differences in both ASDR and ASMR. In Australasia, females exhibited the highest ASDR and ASMR per 100,000 people, with values of 45.88 (95% UI 22.67–69.72) and 2.28 (95% UI 1.09–3.48), respectively (Table S2). Conversely, for males, the highest ASDR was recorded in high-income North America at 61.45 (95% UI 31.08–95.74) per 100,000 people, whereas the highest ASMR was found in Australasia at 2.25 (95% UI 1.12–3.52) per 100,000 people (Table S2). Further analyses of these 21 GBD regions with SDI-based stratification suggested that both ASDR and ASMR tended to initially increase and subsequently decrease with rising SDI levels (Fig. 3A,B).

### Burden of metabolic risk factors for AF/AFL by country

**Fig. 4:**
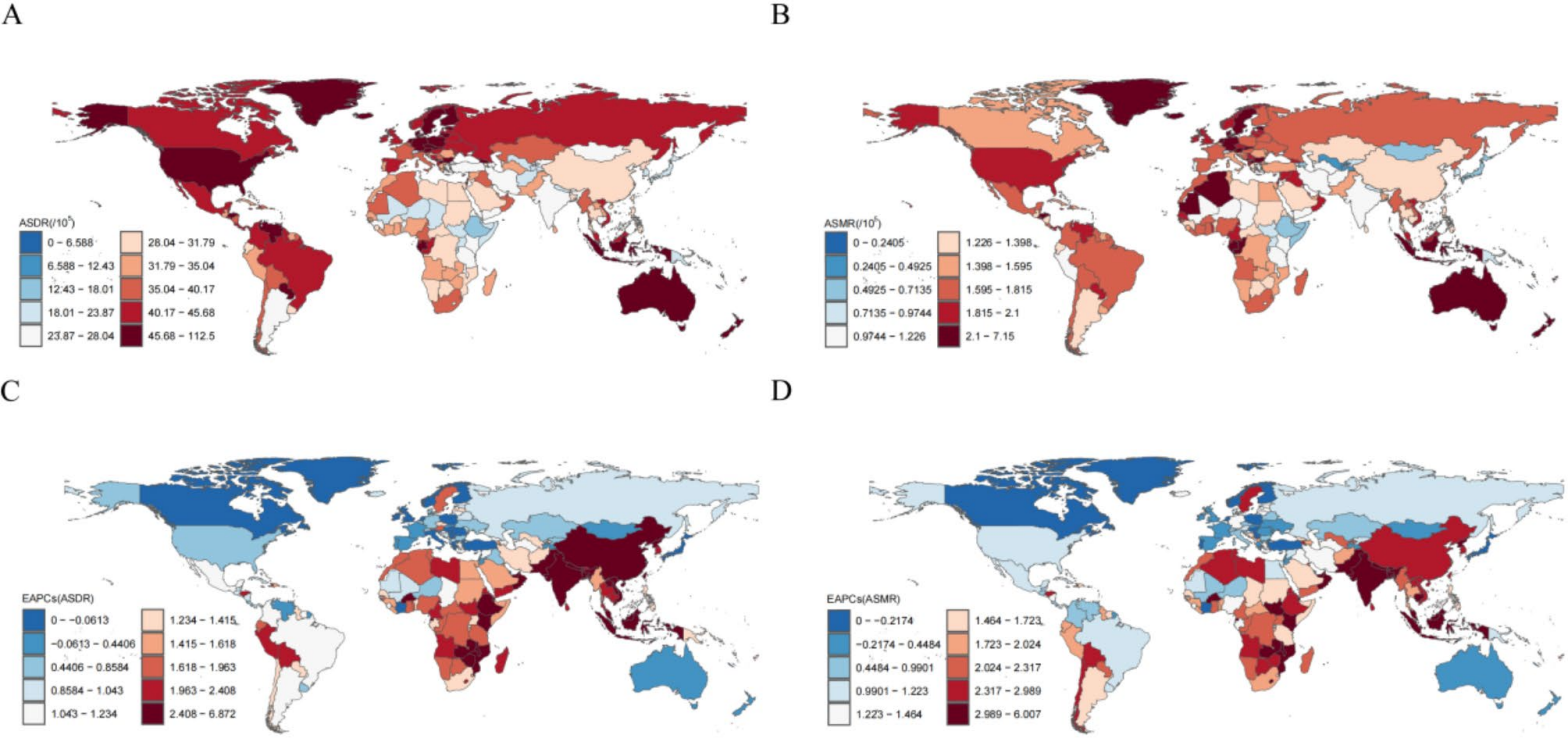
Global AF/AFL burden attributable to metabolic risk factors across 204 countries and territories (**A**) ASDR per 100,000 people in 2021. (**B**) ASMR per 100,000 people in 2021. (**C**) EAPC of DALYs per 100,000 people between 1990 and 2021. (**D**) EAPC of deaths per 100,000 people between 1990 and 2021. AF/AFL = atrial fibrillation /atrial flutter; ASDR = age-standardized rates of disability-adjusted life years; ASMR = age-standardized mortality; and EAPC = estimated annual percentage change.

In both 1990 and 2021, the burden of AF/AFL caused by metabolic risk factors substantially varied across countries. In 2021, the ASMR and ASDR of metabolic risk factors associated with AF/AFL burden varied up to 23-fold between countries. Specifically, the ASMR per 100,000 people ranged from 0.31 (95% UI 0.13–0.52) in Singapore to 7.15 (95% UI 3.54–11.12) in Montenegro (Fig. 4A, Table S3). Similarly, the ASDR per 100,000 people ranged from 12.35 (95% UI 4.97–21.06) in Singapore to 112.49 (95% UI 56.04–173.21) per 100,000 people in Montenegro (Fig. 4B, Table S3).

Between 1990 and 2021, the burden of AF/AFL caused by metabolic risk factors varied significantly across countries. Regarding ASDR, the most substantial increase was observed in Vietnam, with an estimated EAPC of 6.87 (95% UI 0.58–13.56) per 100,000 people (Fig. 4C, Table S3). In contrast, the most substantial decline was observed in Timor-Leste, with an EAPC of − 7.39 (95% UI − 12.34 to − 2.16) per 100,000 people. Comparatively, regarding ASMR, the most substantial increase was recorded in Bangladesh with an EAPC of 6.01 (95% UI 0.23–12.12) per 100,000 people, whereas the most substantial decline was recorded in Timor-Leste with an EAPC of − 5.79 (95% UI − 10.01 to − 1.37) per 100,000 people (Fig. 4D, Table S3).

**Fig. 5:**
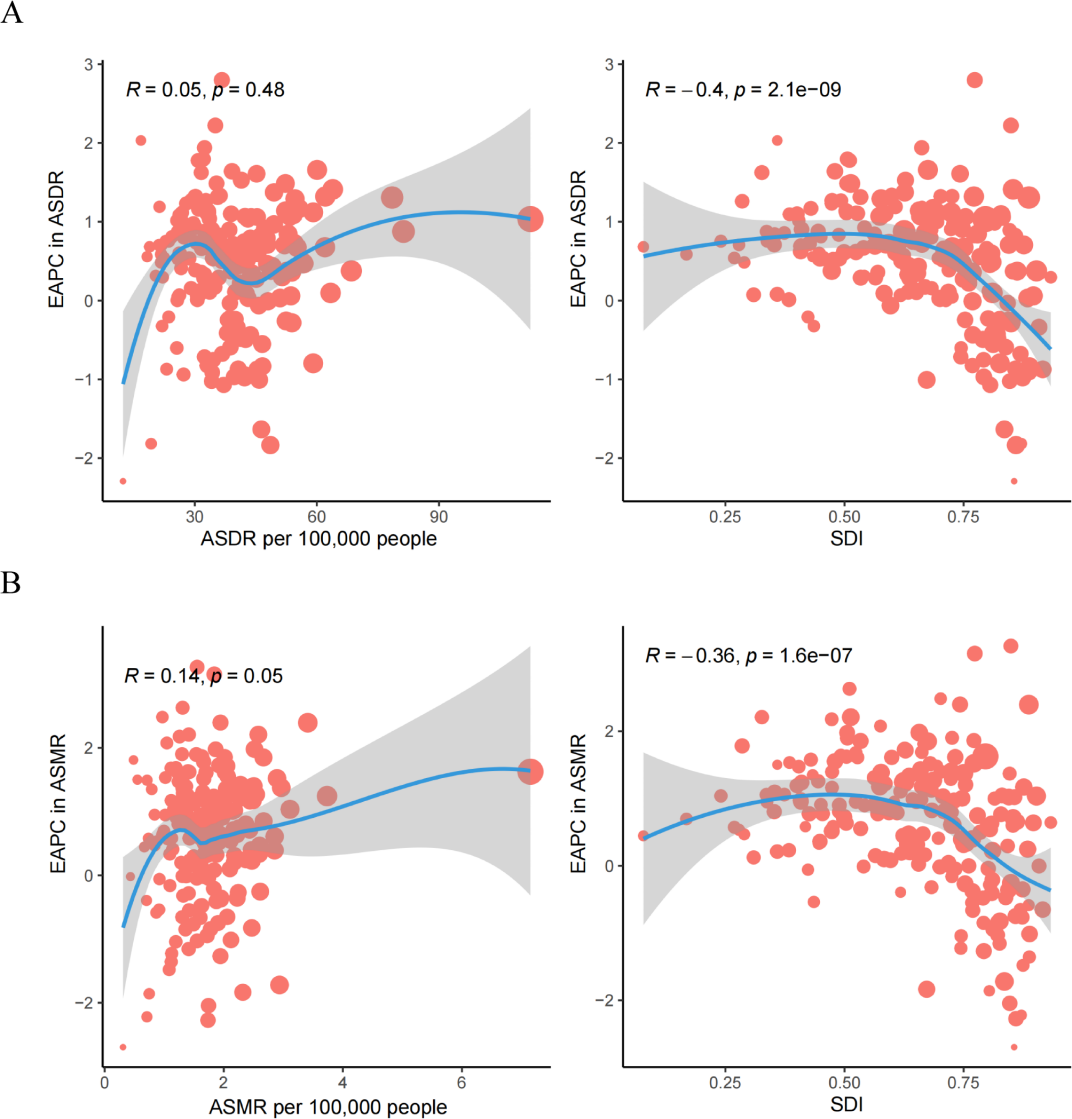
Correlation between EAPC and metabolic risk factors attributable to AF/AFL burden and SDI in 2021 (**A**) EAPC of ASDR per 100,000 people and SDI. (B) EAPC of ASMR per 100,000 people and SDI; the circle size reflects the number of AF/AFL cases. AF/AFL = atrial fibrillation /atrial flutter; ASDR = age-standardized rates of disability-adjusted life years; ASMR = age-standardized mortality; EAPC = estimated annual percentage change; and SDI = socio-demographic index.

In the 2021 analysis of the 204 countries, SDI alone exhibited a unique trend: an overall, albeit gradual increase, was observed in both ASMR and ASDR for AF/AFL attributable to metabolic risk factors with an increase in SDI. Notably, both ASMR and ASDR in Montenegro and Nauru were significantly higher than anticipated based on their SDI levels (Figure S2A,B). At the national level in 2021, the EAPC in ASDR exhibited a significant negative correlation with SDI (r = -0.4, *p* < 0.001). However, there was no significant correlation between the EAPC of ASDR and ASDR itself (Fig. 5A). Similarly, the EAPC in ASMR was negatively correlated with SDI (r = -0.36, *p* < 0.001) but not with ASMR itself (Fig. 5B).

### Burden of metabolic risk factors for AF/AFL projections up to 2030

**Fig. 6:**
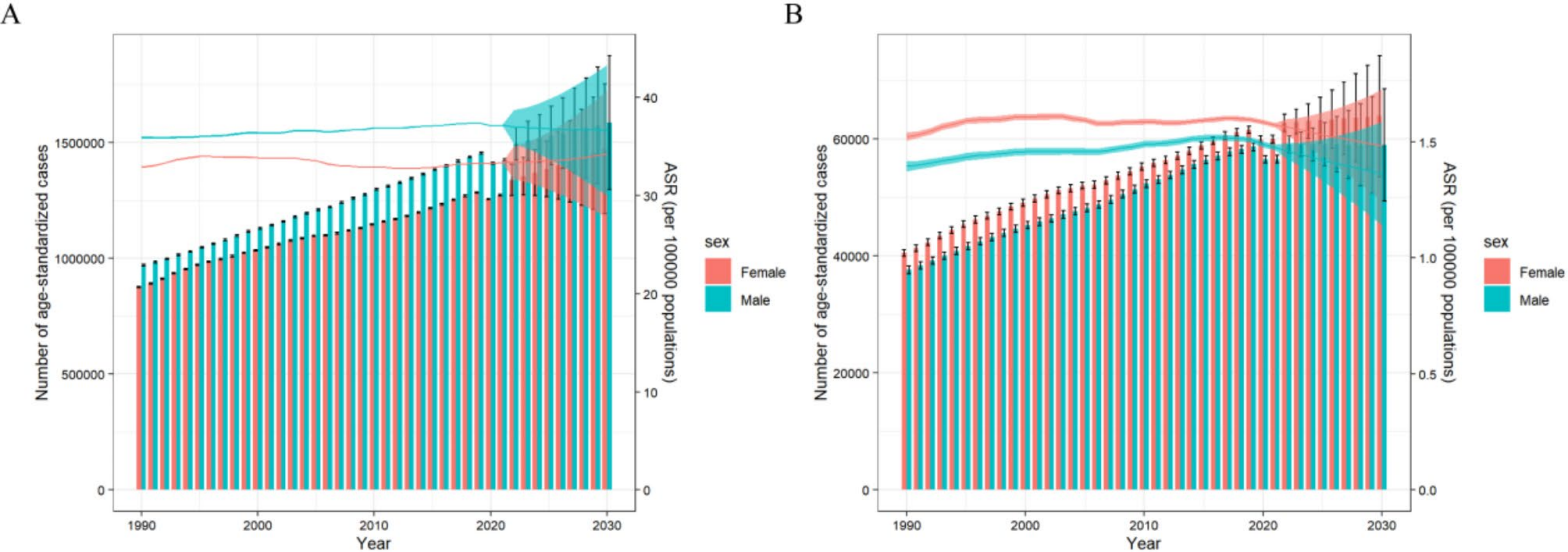
Future trends in metabolic risk factors associated with AF/AFL burden (**A**) The number of DALYs and ASDR per 100,000 people by sex. (**B**) The number of deaths and ASDR per 100,000 people by sex. AF/AFL = atrial fibrillation /atrial flutter; ASDR, age-standardized rates of disability-adjusted life years; ASMR = age-standardized mortality; and DALYs = disability-adjusted life years.

Based on predictions from the BAPC model, the ASMR for AF/AFL associated with metabolic risk factors is expected to trend downward significantly. Concurrently, the ASDR for AF/AFL associated with metabolic risk factors is projected to stabilize with minimal variation. Notably, ASDR will be higher in males than in females, whereas ASMR will be lower in males than in females. More specifically, ASDR is projected to increase marginally from 35.20 (95% UI 33.50–36.90) in 2022 to 35.49 (95% UI 28.91–42.07) in 2030 per 100,000 people (Fig. 6A, Table S4). In contrast, ASMR is projected to decrease from 1.52 (95% UI 1.49–1.56) in 2022 to 1.43 (95% UI 1.21–1.65) in 2030 per 100,000 people (Fig. 6B, Table S4).

## Discussion

AF and AFL present a substantial challenge to public health, owing to rising trends in morbidity and mortality^18^. Maintaining metabolic health–even among people with overweight or obesity without metabolic syndrome-related issues (such as hypertension, diabetes mellitus, and hyperlipidemia)–correlates with a markedly higher likelihood of AF. Therefore, people with overweight or obesity might independently contribute to the risk of AF^19^. Metabolic dysfunction, characterized by the simultaneous presence of hypertension, diabetes mellitus, and hyperlipidemia-is strongly associated with elevated BMI. It is more prevalent among patients with AF, markedly raising the likelihood of all-cause mortality, severe cardiovascular events, and adverse clinical outcomes^19,20^. Consequently, evaluating and controlling metabolic health in patients with AF is crucial for enhancing risk stratification and developing personalized treatment strategies. Elevated SBP, which is a key indicator of metabolic abnormalities, is strongly associated with heightened systemic inflammation and a greater burden on the left atrium, therefore increasing the likelihood of AF^21^. Conversely, a high BMI fosters the onset and advancement of AF via processes, such as fat tissue inflammation, oxidative stress, and abnormalities in the atrial structure^22^.

Despite major research advancements on the AF/AFL burden utilizing the GBD database, most studies have concentrated on examining general or individual risk factors without comprehensively evaluating the collective impact of metabolic risk factors^23–25^. Building upon the GBD 2021 database, this study evaluated the association between metabolic risk factors, particularly high BMI and SBP, and the effects of AF/AFL. This comprehensive perspective addresses the shortcomings of previous research. To the best of our knowledge, this is the first study to utilize the BAPC model to evaluate historical patterns and forecast shifts in the worldwide burden of AF/AFL by 2030. As a pioneering approach, this forward-looking analysis offers vital insights into disease prevention, control, and policy formulation.

Our results reveal a marked increase in the global AF/AFL burden attributable to metabolic risk factors between 1990 and 2021. Notably, a significant rising pattern in ASDR and ASMR associated with these risk factors was observed. The ASDR per 100,000 people increased from 34.22 in 1990 to 34.94 in 2021, comparable to the ASMR trend. These results highlight the growing influence of metabolic risk factors on the worldwide prevalence and severity of AF/AFL.Furthermore, this research uncovered notable sex-related disparities in AF/AFL prevalence, with females experiencing a greater disease burden than males. This disparity was evident in both the number of DALYs and fatalities, with a notably higher prevalence in females. The origin of this sex disparity may be attributed to unique physiological traits in females. For example, their vascular and heart physiology may heighten their vulnerability to atrial alterations and electrophysiological disorders caused by hypertension, increasing their likelihood of developing AF/AFL^26^. Furthermore, females are more susceptible to central obesity, which considerably impacts the atrial structure and cardiac electrical function^27^. A reduction in estrogen levels after menopause may diminish the protective effect of the cardiovascular system, intensifying the impact of metabolic imbalances on the atrial architecture and function^28^.

Furthermore, these findings suggest that the impact of AF/AFL increases with age, peaking in the 80 to 84 years age bracket for DALYs and the 85 to 89 years age bracket for mortality. The worldwide growth in the older adult population is exacerbating the prevalence of AF/AFL, considering the higher propensity for morbidity and metabolic risks in this population. Age-related physiological alterations, including heightened arterial rigidity^29^, intensified inflammation^30^, and changes in heart structure^31^, may make older adults more susceptible to AF and AFL. These results underscore the need for age-specific strategies for preventing and managing AF/AFL. Customized strategies addressing the distinct requirements and obstacles faced by older adults may alleviate the effects of AF/AFL in this high-risk group.

This study underscores significant geographic and socioeconomic disparities in the impact of metabolic risk-related AF/AFL. High-SDI regions, including Australasia, Western Europe, and North America, showed a general decrease in ASDR and ASMR between 1990 and 2021. By contrast, low-to-medium SDI regions exhibited increasing trends. This disparity reflects the intricate interplay between lifestyle habits and genetic predisposition^32^. In high SDI regions, the intensifying impact of genetic predisposition on metabolic risks has been mitigated through healthier lifestyle promotion habits and the use of precision medical technologies. In these areas, enhanced genetic screening and tailored treatments have facilitated early detection and focused care for people at elevated genetic risk, substantially lowering the prevalence of AF/AFL^33^. However, in low-to-medium SDI regions, limited healthcare facilities and unhealthy living patterns attributed to rapid urbanization exacerbate the influence of genetic predisposition. The absence of structured health education and preventative strategies^25^, along with the challenges in obtaining timely interventions, contributes to the rising prevalence of AF/AFL.

The BAPC model forecasts a stabilization in ASDR and a moderate reduction in ASMR for AF/AFL attributable to metabolic risk factors by 2030. While these forecasts are promising, despite a decrease in mortality, the overall disease burden will remain substantial. The predicted stabilization of ASDR, particularly in males, indicates that the global fight against metabolic risk factors is likely to continue. This necessitates ongoing worldwide endeavors to diminish metabolic risk factors via public health programs, health education initiatives, and enhanced healthcare access in low- and medium-SDI regions.

The impact of the coronavirus disease 2019 (COVID-19) pandemic should be considered when evaluating the role of metabolic risk factors in the worldwide burden of AF/AFL. A systematic review of five observational studies involving 19,478,173 patients suggested that patients recuperating from COVID-19 demonstrated a markedly higher likelihood of developing new-onset AF, with a 57% higher risk than patients without COVID-19 (HR 1.57; 95% CI, 1.24–1.99)^34^. Additionally, atrial arrhythmias accounted for 81.8% of all arrhythmias among hospitalized patients with COVID-19^35^. Concurrently, the COVID-19 pandemic significantly influenced the worldwide obesity crisis. A retrospective analysis focusing on American adults reported a notable rise in average BMI during the pandemic (+ 0.6%, *p* < 0.05) and a 3% surge in obesity prevalence (*p* < 0.05). These trends were concurrent with lifestyle changes, such as increased physical activity and sleep duration, as well as reduced smoking rates^36^. Such behavioral changes may have contributed to BMI increase and the buildup of metabolic risk factors. Furthermore, hypertension cases substantially increased throughout the COVID-19 pandemic. One study reported a rise in new hypertension cases from 2.11 per 100 person-years before the pandemic to 5.20 per 100 person-years during the pandemic (RR = 2.46)^37^. The COVID-19 crisis disrupted in-person healthcare services, particularly for patients with arrhythmia, leading to the postponement or cancellation of numerous follow-up appointments and interventions. This resulted in a surge of atrial fibrillation cases after infection^38^. Thus, the COVID-19 pandemic has influenced lifestyle habits, leading to higher rates of hypertension and elevated BMI, thereby intensifying the burden of AF/AFL attributable to metabolic risk factors.

While considering the crucial role of metabolic risk factors in the worldwide impact of AF/AFL, effective management of these factors is necessary to enhance patient outcomes. The Atrial fibrillation Better Care (ABC) integrated management strategy—which emphasizes systematic and process-oriented approaches—focuses on three primary areas: (i) anticoagulation and avoid stroke; (ii) better symptom management; and (iii) cardiovascular risk and comorbidity management. This strategy aims to decrease the risk of ischemic stroke risk via oral anticoagulation while addressing AF, the predominant cause of stroke. Furthermore, it encompasses the thorough treatment of psychological conditions, both cardiovascular and non-cardiovascular risks, and other comorbidities in patients with AF^39^.The Cohort of Antithrombotic Use and Optimal International Normalized Ratio Level in Patients With Non-Valvular Atrial Fibrillation in Thailand registry research followed 3405 patients (average age 67.8 years; 41.8% female) over a period of 3 years. It reported that strict adherence to the ABC pathway successfully managed metabolic disorders, such as hypertension and obesity, markedly decreasing the likelihood of overall mortality and cardiovascular events^40^. Further evidence from the Mobile Health Technology for Improved Screening and Optimized Integrated Care in AF randomized study suggests that integrating the ABC pathway with mobile health technology enhanced patient compliance with treatment and markedly diminished the negative effects of metabolic disorders^41^. Specifically, adherence to the ABC pathway improved outcomes in high-risk patient subgroups, particularly patients with advanced age, chronic kidney disease, and a history of thrombotic incidents^42^. Thus, controlling metabolic risk factors in patients with AF/AFL can markedly enhance their health outcomes through patient-centered strategies incorporated in the ABC pathway.

The escalating worldwide impact of AF/AFL, which is attributed to metabolic risk factors, carries major implications for public health. The mentioned trends, including the escalating prevalence in low-to-medium SDI regions, indicate that existing public health strategies are insufficient to curb the proliferation of metabolic risk factors among these groups. For high-risk groups, particularly older females, regular screenings for metabolic risk factors and prompt action to avert AF/AFL should be prioritized. Considering the association between metabolic risk factors and AF/AFL, strategies aimed at reducing high BMI and high SBP rates are expected to significantly lower the prevalence of AF/AFL.

### Study limitations

The research is subject to multiple limitations, such as potential biases in data collection and the intrinsic limitations of the GBD database. The GBD database is founded on diverse data sources, which can differ in quality and thoroughness^43^. Moreover, the cross-sectional nature of this study restricts the ability to establish causal associations. Longitudinal studies are required to validate these results and investigate long-term trends in the burden of AF/AFL. Extended research can elucidate the temporal association between metabolic risk factors and AF/AFL, facilitating the identification of underlying causes and the development of preventive approaches.

## Conclusion

In conclusion, a notable global rise in the burden of AF/AFL attributable to metabolic risk factors has been reported between 1990 and 2021. This study highlights the implication of managing metabolic risk factors to reduce the prevalence and intensity of AF/AFL. Holistic public health tactics and specific measures are warranted to address this escalating health issue, particularly in areas experiencing rising disease prevalence. Additional studies are crucial for developing and implementing successful strategies to lessen the effects of these conditions on global health. Grasping the intricate interplay between metabolic risk factors and AF/AFL may guide the development of more effective prevention and treatment approaches, thereby enhancing global cardiovascular health.

## Electronic supplementary material

Below is the link to the electronic supplementary material.

Supplementary Material 1

## Data Availability

All data downloaded from GBD database(https://www.healthdata.org/research-analysis/gbd) Further inquiries can be directed to the corresponding author.

## Abbreviations

AF/AFL: Atrial fibrillation/atrial flutter
ASDR: Age-standardized disability-adjusted life years rate
ASMR: Age-standardized mortality rate
EAPC: Estimated annual percentage change
GBD: Global burden of disease
SDI: Socio-demographic index
BAPC: Bayesian age-period-cohort
DALYs: Disability-adjusted life years
YLLs: Years of life lost
YLDs: Years lived with disability
SBP: Systolic blood pressure
BMI: Body mass index
COVID-19: Coronavirus disease 2019
ABC: Atrial fibrillation better care
UI: Uncertainty interval
CI: Confidence interval
HR: Hazard ratio
RR: Risk ratio
