# Supplementary material for: Global trends and epidemiological impact of metabolic risk factors on atrial fibrillation and atrial flutter from 1990 to 2021
Source: Sci Rep. 2025 Feb 7;15:4561. doi: 10.1038/s41598-025-88744-4 (PMC11802920; doi:10.1038/s41598-025-88744-4)

SUPPLEMENTAL MATERIAL

Table S1. The number of DALYs and Deaths attributable to metabolic risk-related AF/AFL by sex and age in 2021.

Abbreviations: AF/AFL, atrial fibrillation /atrial flutter; DALYs, disability-adjusted life years.

Age	Female		Male	
	DALYs(95%UI)	Deaths(95%UI)	DALYs(95%UI)	Deaths(95%UI)
30-34 years	685.40(345.91-1182.29)	8.14(3.97-13.16)	1397.20(600.45-2416.39)	15.55(6.96-25.80)
35-39 years	2399.00(1031.06-4716.88)	15.07(7.49-23.86)	4667.47(1775.68-8886.58)	25.37(11.40-42.01)
40-44 years	6982.84(3011.22-12703.49)	46.76(23.53-73.7)	12653.28(5381.31-23620.73)	71.99(33.43-113.68)
45-49 years	17038.73(7145.29-30470.29)	127.91(61.9-201.0)	27665.03(12123.80-47967.45)	181.34(84.24-281.66)
50-54 years	34732.10(15663.22-60920.08)	307.10(149.41-465.52)	53750.94(22628.29-96786.83)	436.28(194.31-687.14)
55-59 years	63231.17(29769.00-109971.84)	708.39(343.46-1076.18)	88904.05(37540.45-150951.77)	860.80(376.02-1324.38)
60-64 years	93794.98(44265.07-158249.09)	1241.27(589.14-1867.03)	124835.38(54066.25-210513.70)	1443.17(643.89-2182.77)
65-69 years	142776.55(66261.87-227923.40)	2213.63(1030.01-3294.61)	165952.06(71484.82-270885.17)	2239.87(984.83-3378.97)
70-74 years	198537.27(89991.63-310043.15)	4450.39(2095.15-6700.63)	201690.95(85479.93-328222.75)	3946.36(1717.18-6111.17)
75-79 years	217373.01(97810.67-340795.88)	6973.31(3212.54-10524.7)	194854.37(84049.89-313506.36)	5482.27(2442.26-8517.25)
80-84	279428.29(121942.84-434281.81)	14218.57(6409.63-22050.64)	204846.91(88936.52-329023.77)	9447.74(4024.57-14883.98)
85-89	240431.42(105447.30-374748.39)	17503.22(7672.95-27335.52)	145187.52(61402.8-230227.78)	9826.60(4232.90-15212.61)
90-94	180040.50(80754.43-291685.68)	17389.41(7790.06-27640.66)	80596.91(35327.88-128633.81)	7402.35(3353.79-11787.61)
95+ years	71378.74(32077.26-115927.92)	7737.58(3466.7-12594.77)	25968.40(11677.64-42183.38)	2692.04(1192.21-4332.00)

Table S2. The burden of metabolic risk-related AF/AFL across 21 Global Burden of Disease regions, stratified by sex, in the year 2021.

Abbreviations: AF/AFL, atrial fibrillation /atrial flutter; ASDR, age-standardized rates of disability-adjusted life years; ASMR,age-standardized mortality rate.

Region	Female		Male	
	ASDR(95% UI)	ASMR(95%UI)	ASDR(95%UI)	ASMR(95%UI)
Andean Latin America	30.46(14.01-47.40)	1.30(0.59-2.07)	34.07(14.51-54.99)	1.22（0.52-2.01）
Australasia	45.88(22.67-69.72)	2.28(1.09-3.48)	59.62(27.31-93.23)	2.25(1.12-3.52)
Caribbean	35.79(16.54-55.70)	1.55(0.71-2.44)	40.87(17.77-65.86)	1.58(0.68-2.51)
Central Asia	27.19(13.27-40.65)	0.94(0.47-1.37)	33.04(13.84-52.32)	0.97(0.42-1.47)
Central Europe	38.75(19.22-57.37)	1.72(0.88-2.53)	51.40(23.71-77.96)	1.83(0.85-2.74)
Central Latin America	40.57(19.60-61.18)	1.72(0.83-2.54)	45.45(20.21-69.75)	1.67(0.75-2.56)
Central Sub-Saharan Africa	31.95(11.27-58.75)	1.53(0.49-3.07)	33.35(11.36-54.89)	1.54(0.49-2.74)
East Asia	28.75(11.44-48.00)	1.47(0.57-2.55)	27.41(10.11-46.34)	1.14(0.41-1.92)
Eastern Europe	41.94(21.18-62.28)	1.82(0.94-2.59)	46.95(21.30-72.95)	1.64(0.78-2.45)
Eastern Sub-Saharan Africa	23.74(8.72-42.44)	0.98(0.3-1.88)	23.60(7.83-41.65)	0.93(0.28-1.89)
High-income Asia Pacific	13.82(5.10-23.40)	0.56(0.2-0.95)	26.96(9.81-45.06)	0.93(0.36-1.54)
High-income North America	42.85(22.35-64.47)	1.74(0.88-2.62)	61.45(31.08-95.74)	2.08(1.04-3.22)
North Africa and Middle East	33.60(18.20-49.55)	1.92(0.99-2.85)	24.35(11.96-37.79)	0.99(0.49-1.52)
Oceania	31.78(14.78-50.20)	1.26(0.56-2.02)	27.94(11.61-45.61)	0.99(0.37-1.73)
South Asia	25.62(9.02-43.79)	1.04(0.33-1.87)	28.47(9.73-47.28)	1.20(0.42-2.06)
Southern Latin America	26.24(12.58-39.36)	1.43(0.68-2.14)	32.96(15.79-50.43)	1.36(0.63-2.06)
Southern Sub-Saharan Africa	38.64(19.91-57.44)	1.75(0.84-2.60)	35.22(16.52-54.05)	1.37(0.64-2.07)
Southeast Asia	41.24(16.30-66.57)	1.96(0.76-3.15)	36.78(13.12-60.63)	1.52(0.56-2.55)
Tropical Latin America	40.17(18.88-62.17)	1.72(0.79-2.59)	49.01(21.71-77.53)	1.75(0.79-2.71)
Western Europe	39.35(18.93-60.26)	1.97(0.94-3.00)	55.42(25.61-87.63)	1.96(0.87-3.02)
Western Sub-Saharan Africa	31.74(13.40-49.61)	1.66(0.68-2.62)	32.2(12.89-52.13)	1.61(0.67-2.77)

Table S3. The burden of AF/AFL associated with metabolic risk factors across 204 countries.

Abbreviations: AF/AFL, atrial fibrillation /atrial flutter; ASDR, age-standardized rates of disability-adjusted life years; ASMR,age-standardized mortality rate; EAPC,estimated annual percentage change.

Country	ASMR(95% UI)	ASDR(95%,UI)	EAPC(95%,UI)	
			ASMR	ASDR
Montenegro	7.15 (3.54-11.12)	112.49 (56.04-173.21)	1.93(0.80-3.08)	1.30(0.25-2.36)
Nauru	3.73 (1.57-7.73)	81.28 (40.91-134.74)	1.71(0.44-3.00)	1.14(0.17-2.13)
Sweden	3.41 (1.51-5.52)	78.5 (35.06-125.54)	2.78(1.02-4.57)	1.73(0.09-3.41)
Germany	3.11 (1.46-4.72)	68.48 (32.21-105.01)	1.25(-0.22-2.75)	0.66(-0.75-2.08)
Austria	2.97 (1.32-4.78)	63.9 (28.83-100.04)	1.02(-0.52-2.59)	1.71(0.17-3.28)
Slovakia	2.94 (1.24-4.8)	63.37 (31.68-94.99)	0.42(-0.70-1.55)	0.27(-0.79-1.34)
Northern Mariana Islands	2.89 (1.48-4.38)	62.07 (32.21-92.39)	1.83(0.71-2.97)	1.51(0.62-2.41)
Niue	2.85 (1.34-4.43)	61.98 (29.32-93.64)	1.25(-0.21-2.74)	1.07(-0.09-2.25)
Fiji	2.85 (1.48-4.34)	60.05 (30.24-90.03)	2.13(1.12-3.14)	1.88(0.96-2.79)
American Samoa	2.67 (1.25-4.49)	59.11 (30.6-90.45)	1.64(0.62-2.67)	1.27(0.47-2.08)
Greenland	2.67 (1.3-4.02)	59.1 (28.34-94.12)	-0.58(-1.78-0.64)	-0.56(-1.64-0.53)
Dominica	2.61 (1.2-4.04)	56.78 (28.98-87.49)	0.74(-0.32-1.80)	0.77(-0.19-1.74)
Israel	2.6 (1.11-4.24)	56.45 (25.61-87.79)	-0.16(-1.61-1.31)	0.77(-0.63-2.18)
Tokelau	2.59 (1.14-4.0)	55.56 (25.42-88.85)	1.72(0.38-3.07)	1.42(0.38-2.48)
North Macedonia	2.58 (1.09-4.21)	55.1 (25.93-86.41)	1.83(0.42-3.25)	1.03(-0.26-2.34)
Tuvalu	2.55 (1.12-4.36)	54.62 (27.54-84.27)	1.94(0.36-3.55)	1.58(0.44-2.73)
Micronesia (Federated States of)	2.52 (0.97-4.06)	54.48 (28.05-86.07)	1.39(0.07-2.73)	1.02(0.11-1.94)
Samoa	2.5 (1.2-4.11)	54.41 (28.27-84.25)	1.21(-0.02-2.45)	0.95(0.06-1.85)
Czechia	2.49 (1.19-3.89)	54.17 (26.54-82.97)	0.98(-0.21-2.19)	1.26(0.17-2.36)
New Zealand	2.47 (1.23-3.7)	53.86 (25.1-83.98)	0.22(-1.16-1.61)	0.21(-1.05-1.49)

Bulgaria	2.47 (1.17-3.91)	53.79 (26.19-82.45)	0.62(-0.58-1.84)	0.55(-0.61-1.73)
Denmark	2.46 (1.16-3.68)	53.61 (22.77-85.98)	1.14(-0.69-3.01)	0.54(-1.20-2.32)
Marshall Islands	2.46 (1.09-4.05)	53.36 (25.39-82.56)	1.43(0.31-2.56)	1.25(0.37-2.13)
Cook Islands	2.44 (1.13-3.75)	52.93 (26.27-83.17)	0.90(-0.15-1.97)	0.94(0.12-1.77)
United States of America	2.43 (1.12-3.84)	52.62 (27.53-80.35)	1.18(0.03-2.34)	0.84(-0.11-1.81)
Australia	2.37 (1.11-3.59)	52.44 (24.55-80.19)	0.34(-0.92-1.62)	0.39(-0.79-1.57)
Indonesia	2.37 (1.13-3.83)	52.29 (19.75-83.85)	5.29(-0.04-10.90)	3.65(-0.74-8.24)
Honduras	2.32 (0.92-3.82)	52.26 (23.75-84.54)	2.92(1.09-4.79)	2.04(0.42-3.68)
Iceland	2.28 (1.09-3.64)	52.04 (23.61-79.99)	1.46(0.08-2.86)	0.94(-0.35-2.26)
Saint Kitts and Nevis	2.28 (0.98-3.6)	51.46 (24.06-81.42)	1.21(-0.50-2.94)	0.99(-0.54-2.54)
Luxembourg	2.25 (1.11-3.66)	50.97 (24.14-78.16)	0.66(-0.69-2.02)	0.40(-0.95-1.77)
Tonga	2.24 (0.79-3.92)	49.46 (26.21-76.79)	2.08(1.02-3.15)	1.55(0.78-2.33)
Georgia	2.23 (1.08-3.43)	49.43 (22.32-75.69)	1.70(0.21-3.21)	1.12(-0.26-2.52)
Gabon	2.22 (0.93-3.48)	49.07 (22.11-76.23)	1.70(0.21-3.21)	1.12(-0.26-2.52)
Vanuatu	2.19 (1.07-3.28)	48.66 (20.14-76.77)	1.45(-0.52-3.45)	1.25(-0.49-3.01)
Finland	2.18 (1.01-3.42)	48.57 (22.65-76.5)	1.43(-1.24-4.17)	1.16(-1.03-3.39)
Trinidad and Tobago	2.17 (0.88-3.48)	47.97 (23.09-75.47)	-1.79(-3.243 to -0.315)	-1.38(-2.82-0.09)
Estonia	2.17 (0.97-3.51)	47.8 (23.11-70.68)	0.12(-1.42-1.68)	0.42(-0.99-1.86)
Bahamas	2.15 (1.0-3.48)	47.74 (22.76-72.93)	1.23(-0.01-2.48)	0.91(-0.25-2.09)
Paraguay	2.12 (0.95-3.33)	47.63 (21.24-75.72)	1.14(-0.32-2.62)	0.95(-0.34-2.25)
Barbados	2.11 (0.98-3.31)	47.27 (22.16-73.38)	2.13(0.36-3.93)	1.38(-0.12-2.89)
Grenada	2.1 (0.78-3.42)	47.14 (21.25-74.38)	1.57(0.04-3.13)	1.38(0.03-2.75)
Netherlands	2.07 (0.88-3.36)	46.62 (20.41-73.36)	2.31(0.12-4.53)	1.61(-0.21-3.46)
Poland	2.07 (0.87-3.12)	46.56 (21.91-71.76)	-0.56(-2.22-1.12)	-0.40(-2.04-1.26)
Cyprus	2.06 (0.71-3.48)	46.35 (20.78-73.45)	-0.63(-1.88-0.65)	-0.27(-1.51-0.99)
Lithuania	2.06 (0.87-3.26)	46.11 (22.72-69.57)	-0.79(-2.64-1.09)	-0.79(-2.57-1.02)

Saint Lucia	2.04 (0.83-3.37)	45.82 (19.13-75.29)	1.35(0.15-2.57)	1.14(-0.06-2.35)
Venezuela	2.02 (0.93-3.03)	45.7 (21.41-72.12)	-0.51(-2.86-1.89)	0.02(-1.95-2.02)
Russian Federation	2.02 (0.79-3.35)	45.43 (21.94-68.84)	0.63(-0.80-2.07)	0.44(-0.85-1.75)
Norway	2.01 (0.94-3.28)	45.37 (19.62-71.76)	1.00(-0.19-2.21)	0.98(-0.18-2.15)
Latvia	1.99 (0.89-3.23)	45.29 (21.75-68.56)	-0.29(-2.13-1.58)	-0.50(-2.27-1.30)
Panama	1.98 (0.94-3.14)	45.27 (22.31-70.07)	1.25(0.06-2.46)	1.25(0.10-2.40)
Malaysia	1.96 (0.85-3.08)	45.18 (18.77-71.15)	1.13(0.07-2.20)	0.97(-0.04-1.98)
Antigua and Barbuda	1.95 (0.91-3.25)	45.06 (20.66-70.82)	3.75(0.98-6.59)	2.46(0.17-4.79)
El Salvador	1.95 (0.81-2.99)	44.95 (21.56-71.11)	1.17(-0.62-3.00)	0.94(-0.64-2.56)
Republic of Moldova	1.94 (0.94-2.86)	44.67 (21.98-66.04)	1.39(0.06-2.73)	1.10(-0.10-2.31)
Brazil	1.93 (0.98-2.92)	44.07 (20.25-68.93)	0.21(-0.90-1.34)	0.89(-0.19-1.99)
Palau	1.92 (0.94-3.22)	43.85 (21.53-65.96)	1.22(-0.38-2.85)	1.04(-0.40-2.51)
Mexico	1.92 (0.77-3.12)	43.67 (20.24-66.09)	1.41(0.33-2.50)	1.18(0.30-2.08)
Spain	1.91 (0.74-3.1)	43.67 (20.81-68.58)	1.06(-0.31-2.45)	1.20(0.01-2.42)
Saint Vincent and the Grenadines	1.9 (0.92-2.88)	43.51 (18.6-69.88)	-0.21(-1.48-1.07)	-0.01(-1.22-1.22)
Costa Rica	1.9 (0.99-2.81)	43.06 (20.09-66.69)	1.58(-1.01-4.24)	1.35(-0.88-3.64)
Jamaica	1.89 (0.87-2.96)	43.03 (19.77-67.62)	0.66(-0.97-2.32)	0.58(-0.89-2.08)
Seychelles	1.88 (0.95-2.86)	42.97 (19.25-69.89)	1.63(-0.02-3.31)	1.62(0.22-3.04)
Belarus	1.88 (0.87-2.83)	42.92 (20.7-66.62)	2.43(0.19-4.73)	1.44(-0.34-3.26)
Congo	1.87 (0.89-2.85)	42.59 (17.0-67.62)	0.88(-0.42-2.19)	0.92(-0.33-2.19)
Hungary	1.86 (0.81-3.0)	42.47 (21.18-63.36)	2.17(-0.96-5.39)	1.62(-0.96-4.28)
Equatorial Guinea	1.86 (0.89-2.8)	42.33 (17.95-67.16)	-0.22(-1.47-1.05)	-0.25(-1.40-0.91)
Ireland	1.86 (0.93-2.8)	42.21 (18.55-65.92)	3.08(0.65-5.57)	2.42(0.28-4.62)
Guyana	1.84 (0.94-2.9)	42.2 (18.62-68.21)	0.23(-1.33-1.81)	-0.40(-1.89-1.11)
Canada	1.84 (0.84-2.91)	42.07 (19.54-65.6)	1.81(-0.20-3.85)	1.38(-0.36-3.14)

Albania	1.83 (0.65-3.15)	41.79 (18.32-63.39)	-0.41(-1.61-0.80)	-0.54(-1.62-0.56)
Belize	1.83 (0.7-3.1)	41.47 (20.09-64.13)	1.32(-0.05-2.72)	0.79(-0.58-2.17)
Viet Nam	1.82 (0.85-3.0)	41.43 (14.67-67.96)	1.27(0.09-2.46)	0.97(-0.07-2.02)
United States Virgin Islands	1.82 (0.81-2.97)	41.22 (19.24-64.76)	2.93(-3.58-9.89)	6.87(0.58-13.56)
Ukraine	1.82 (0.73-2.89)	41.19 (19.57-61.71)	-0.68(-1.66-0.31)	-0.33(-1.25-0.60)
Haiti	1.81 (0.91-2.61)	40.71 (14.09-69.32)	0.22(-0.91-1.37)	0.46(-0.64-1.57)
United Kingdom	1.8 (0.8-2.95)	40.66 (19.85-62.35)	2.28(-1.66-6.38)	2.13(-1.40-5.79)
Colombia	1.78 (0.88-2.93)	40.64 (18.32-62.47)	-0.07(-1.45-1.33)	-0.24(-1.53-1.06)
Monaco	1.76 (0.87-2.68)	40.41 (18.31-62.73)	0.79(-0.91-2.53)	1.08(-0.49-2.68)
Bosnia and Herzegovina	1.76 (0.72-2.76)	40.19 (19.41-62.08)	0.31(-1.02-1.66)	-0.05(-1.31-1.24)
Mauritius	1.75 (0.64-3.05)	39.98 (16.97-62.43)	1.47(0.02-2.94)	0.94(-0.43-2.32)
Puerto Rico	1.74 (0.51-3.13)	39.96 (19.24-61.21)	0.95(-1.39-3.35)	0.84(-1.24-2.96)
Serbia	1.74 (0.73-2.8)	39.66 (19.7-58.53)	-0.81(-1.96-0.35)	0.07(-0.94-1.09)
Iraq	1.74 (0.81-2.62)	39.46 (18.92-62.19)	-0.61(-2.06-0.86)	-0.38(-1.75-1.01)
Cameroon	1.73 (0.83-2.64)	39.14 (17.82-59.83)	1.31(0.24-2.39)	0.83(-0.21-1.88)
Belgium	1.72 (0.79-2.64)	38.95 (17.34-60.62)	2.06(0.27-3.87)	2.05(0.39-3.74)
Eswatini	1.72 (0.86-2.62)	38.94 (20.12-57.57)	1.05(-0.56-2.68)	0.77(-0.79-2.36)
France	1.7 (0.69-2.86)	38.75 (18.09-59.42)	1.56(0.67-2.46)	1.47(0.55-2.39)
Slovenia	1.7 (0.67-2.7)	38.63 (18.59-58.54)	0.20(-1.44-1.87)	0.07(-1.51-1.68)
Bermuda	1.69 (0.55-3.09)	38.55 (18.09-59.57)	0.55(-0.64-1.75)	0.19(-0.93-1.32)
Malta	1.69 (0.81-2.5)	38.54 (16.94-59.96)	-0.48(-1.75-0.81)	0.07(-1.06-1.21)
South Africa	1.68 (0.73-2.63)	38.44 (18.9-57.26)	0.74(-0.98-2.49)	0.52(-1.14-2.20)
Mauritania	1.68 (0.82-2.53)	38.41 (17.99-59.72)	2.01(0.70-3.33)	1.31(0.12-2.52)
Bahrain	1.67 (0.78-2.55)	38.14 (16.11-62.65)	1.21(-0.18-2.61)	1.04(-0.32-2.43)
Brunei Darussalam	1.66 (0.81-2.47)	37.88 (15.14-62.95)	0.45(-0.82-1.73)	0.39(-0.79-1.58)
Nicaragua	1.66 (0.58-2.82)	37.62 (18.4-58.8)	1.69(-1.80-5.30)	1.20(-1.38-3.84)

Kazakhstan	1.66 (0.66-2.78)	37.54 (17.85-56.46)	1.45(0.03-2.89)	1.00(-0.24-2.27)
Italy	1.65 (0.69-2.66)	37.1 (16.53-58.57)	0.95(-0.28-2.20)	0.70(-0.53-1.94)
Andorra	1.65 (0.59-2.73)	36.73 (15.27-60.36)	0.57(-1.08-2.24)	-0.32(-1.91-1.29)
Dominican Republic	1.65 (0.81-2.53)	36.65 (15.93-59.66)	-0.23(-1.99-1.56)	-0.26(-1.94-1.44)
Oman	1.63 (0.72-2.54)	36.65 (19.37-55.84)	1.72(-1.00-4.51)	1.61(-0.49-3.77)
Bolivia (Plurinational State of)	1.63 (0.77-2.37)	36.14 (15.15-60.1)	3.75(2.38-5.14)	3.34(2.09-4.61)
Algeria	1.62 (0.67-2.63)	35.98 (17.59-56.57)	2.54(0.53-4.59)	2.00(0.39-3.65)
Myanmar	1.62 (0.52-2.99)	35.88 (12.88-58.83)	2.53(0.76-4.34)	1.75(0.12-3.40)
Chile	1.62 (0.82-2.32)	35.83 (16.27-53.71)	2.23(-2.63-7.34)	1.52(-2.37-5.55)
Cabo Verde	1.61 (0.55-2.82)	35.59 (15.07-58.49)	2.32(0.84-3.82)	1.24(-0.06-2.56)
Sao Tome and Principe	1.61 (0.75-2.44)	35.32 (14.98-55.6)	3.24(0.57-5.97)	2.36(0.01-4.78)
United Arab Emirates	1.61 (0.71-2.43)	35.04 (17.89-52.01)	2.97(0.62-5.38)	2.23(0.11-4.39)
Senegal	1.6 (0.75-2.56)	34.89 (13.61-55.49)	4.16(2.83-5.49)	3.00(1.85-4.17)
Cuba	1.6 (0.78-2.43)	34.87 (16.38-56.85)	1.71(-1.09-4.59)	1.29(-1.26-3.90)
Syrian Arab Republic	1.6 (0.6-2.77)	34.86 (17.75-54.12)	1.18(-0.62-3.02)	0.97(-0.58-2.55)
Greece	1.59 (0.74-2.51)	34.53 (15.59-53.93)	1.01(-0.06-2.09)	1.12(0.10-2.15)
Lao People's Democratic Republic	1.58 (0.7-2.43)	34.49 (13.14-59.22)	-0.09(-1.65-1.50)	-0.06(-1.51-1.40)
Angola	1.55 (0.73-2.45)	34.4 (12.74-58.01)	2.83(-1.30-7.13)	2.41(-1.26-6.22)
San Marino	1.55 (0.72-2.47)	34.28 (14.84-53.47)	2.88(-1.05-6.98)	2.34(-1.14-5.94)
Togo	1.55 (0.79-2.31)	34.25 (13.59-53.72)	-0.99(-2.41-0.45)	-0.55(-1.90-0.82)
Gambia	1.54 (0.52-3.06)	34.22 (13.78-55.4)	2.01(-0.73-4.83)	1.63(-0.91-4.24)
Guatemala	1.5 (0.68-2.33)	34.19 (16.04-54.22)	2.00(-0.78-4.86)	1.48(-0.94-3.96)
Qatar	1.5 (0.62-2.47)	34.14 (15.64-55.33)	0.45(-0.94-1.87)	1.01(-0.27-2.30)
Suriname	1.5 (0.69-2.46)	33.66 (14.34-54.45)	-1.56(-2.56 to -0.54)	-0.56(-1.51-0.39)
Turkmenistan	1.5 (0.73-2.26)	33.57 (14.74-52.86)	1.48(-1.03-4.05)	1.14(-0.99-3.31)

Sri Lanka	1.49 (0.64-2.59)	33.33 (12.93-54.67)	1.29(-0.26-2.87)	1.12(-0.33-2.59)
Nigeria	1.49 (0.73-2.27)	33.31 (13.46-52.74)	2.86(-0.15-5.96)	2.20(-0.47-4.94)
Guam	1.48 (0.56-2.35)	33.26 (15.84-52.42)	1.34(-0.64-3.36)	1.81(-0.19-3.85)
Pakistan	1.45 (0.56-2.52)	33.21 (12.76-55.12)	-1.65(-2.87 to -0.41)	0.27(-0.81-1.37)
Madagascar	1.44 (0.74-2.22)	33.12 (11.52-58.05)	4.36(0.62-8.24)	3.59(0.27-7.03)
Romania	1.43 (0.44-2.79)	33.04 (16.56-48.86)	2.25(-0.97-5.57)	2.16(-0.89-5.31)
Kiribati	1.43 (0.69-2.14)	32.98 (16.11-52.7)	-0.18(-1.51-1.18)	-0.50(-1.77-0.78)
The Republic of Côte d'Ivoire	1.42 (0.76-2.11)	32.72 (12.91-51.95)	2.19(0.61-3.79)	1.47(0.36-2.59)
Zimbabwe	1.42 (0.65-2.34)	32.71 (13.48-50.73)	1.44(-0.92-3.85)	1.40(-0.86-3.70)
Guinea-Bissau	1.42 (0.54-2.53)	32.7 (12.55-54.38)	2.78(0.24-5.39)	2.47(0.24-4.74)
Morocco	1.42 (0.67-2.08)	32.6 (14.78-50.84)	2.02(-1.09-5.23)	1.64(-1.15-4.51)
Timor-Leste	1.41 (0.66-2.15)	32.51 (11.66-54.4)	2.28(0.22-4.39)	1.79(-0.14-3.76)
Peru	1.41 (0.46-2.63)	32.39 (13.67-52.29)	-5.79(-10.01 to -1.37)	-7.39(-12.34 to -2.16)
Palestine	1.41 (0.69-2.15)	32.38 (16.57-48.38)	1.99(0.32-3.69)	2.15(0.78-3.54)
Portugal	1.41 (0.67-2.2)	32.37 (13.98-51.48)	0.75(-0.17-1.69)	0.72(-0.17-1.62)
Liberia	1.4 (0.46-2.47)	32.22 (13.72-51.93)	-0.49(-2.03-1.09)	-0.05(-1.57-1.51)
Ghana	1.4 (0.52-2.4)	32.19 (13.2-51.36)	1.82(-0.71-4.41)	1.44(-0.63-3.56)
Zambia	1.38 (0.65-2.15)	32.07 (11.61-59.13)	2.08(-0.73-4.97)	1.96(-0.64-4.62)
Croatia	1.37 (0.54-2.3)	31.65 (15.94-48.09)	3.76(0.59-7.03)	3.47(0.73-6.29)
Namibia	1.37 (0.54-2.29)	31.6 (14.41-49.43)	1.11(-0.26-2.50)	1.09(-0.18-2.37)
Democratic People's Republic of Korea	1.36 (0.4-2.71)	31.6 (11.41-53.85)	2.04(0.51-3.60)	1.62(0.11-3.16)
Mozambique	1.36 (0.75-2.06)	31.58 (11.15-54.96)	3.75(0.17-7.45)	3.41(-0.37-7.33)
Philippines	1.36 (0.62-2.15)	31.48 (12.03-53.72)	3.99(-0.21-8.38)	3.12(-0.44-6.81)
Maldives	1.35 (0.66-1.96)	31.37 (11.74-50.89)	1.70(-1.30-4.80)	1.40(-1.22-4.09)
Sierra Leone	1.34 (0.5-2.18)	31.16 (12.06-49.51)	2.30(-2.37-7.19)	1.62(-1.64-5.00)

Solomon Islands	1.34 (0.48-2.33)	30.95 (11.99-54.75)	1.35(-2.13-4.95)	1.06(-2.04-4.26)
Ecuador	1.33 (0.43-2.81)	30.93 (15.15-48.16)	2.26(-0.29-4.88)	1.70(-0.17-3.60)
Egypt	1.32 (0.61-2.11)	30.93 (16.01-45.17)	1.88(0.37-3.41)	1.76(0.60-2.93)
Lesotho	1.32 (0.52-2.26)	30.82 (14.96-47.9)	1.09(0.28-1.90)	1.46(0.67-2.26)
Democratic Republic of the Congo	1.32 (0.47-2.23)	30.62 (10.99-53.19)	3.17(1.71-4.64)	2.22(0.89-3.57)
Central African Republic	1.31 (0.61-2.01)	30.56 (10.81-54.45)	2.19(-0.81-5.26)	1.83(-0.97-4.71)
Botswana	1.31 (0.62-2.09)	30.39 (13.56-47.88)	1.85(-1.89-5.75)	1.65(-1.71-5.13)
Libya	1.31 (0.6-2.09)	30.23 (14.41-49.22)	2.33(0.52-4.17)	1.71(0.07-3.38)
Uruguay	1.31 (0.65-2.06)	30.14 (14.47-47.09)	2.72(1.41-4.05)	2.05(0.84-3.27)
Sudan	1.3 (0.7-1.94)	30.1 (14.32-47.07)	1.16(-0.25-2.60)	0.74(-0.53-2.03)
Armenia	1.3 (0.55-2.14)	30.03 (13.73-47.26)	1.52(-0.12-3.18)	1.53(-0.01-3.11)
Burkina Faso	1.3 (0.56-2.3)	29.66 (10.89-49.89)	2.55(1.29-3.82)	1.42(0.24-2.62)
Saudi Arabia	1.28 (0.43-2.2)	29.65 (15.62-44.27)	3.73(-1.98-9.78)	2.57(-2.30-7.69)
Bangladesh	1.27 (0.42-2.2)	29.09 (9.77-49.97)	1.59(0.59-2.61)	1.51(0.57-2.47)
Thailand	1.26 (0.42-2.28)	29.07 (11.39-48.82)	6.01(0.23-12.12)	4.68(-0.29-9.91)
Kuwait	1.25 (0.48-2.06)	28.79 (15.11-42.68)	1.84(-1.40-5.19)	1.98(-0.77-4.79)
China	1.24 (0.56-2.04)	28.62 (11.41-47.59)	2.08(0.91-3.25)	1.49(0.48-2.50)
Bhutan	1.24 (0.35-2.65)	27.87 (10.54-47.26)	2.65(-1.03-6.46)	3.08(-0.32-6.60)
Djibouti	1.24 (0.41-2.27)	27.33 (9.39-48.08)	3.22(-0.12-6.68)	2.11(-0.58-4.87)
Tunisia	1.23 (0.52-2.02)	27.3 (13.2-45.55)	3.08(-2.19-8.63)	2.57(-2.08-7.44)
Taiwan (Province of China)	1.2 (0.52-2.11)	27.23 (11.73-44.48)	2.42(0.75-4.12)	1.84(0.32-3.39)
Azerbaijan	1.2 (0.51-1.97)	26.97 (11.9-43.43)	0.12(-2.22-2.52)	0.45(-1.75-2.71)
Kenya	1.2 (0.58-1.83)	26.88 (10.14-44.45)	1.73(0.37-3.10)	1.21(-0.02-2.46)
Cambodia	1.16 (0.39-1.97)	26.62 (9.08-45.96)	3.30(-0.10-6.82)	2.47(-0.42-5.45)
Malawi	1.14 (0.56-1.78)	26.5 (9.45-44.07)	3.27(-2.06-8.90)	1.98(-2.29-6.43)

United Republic of Tanzania	1.12 (0.51-1.74)	26.4 (10.08-45.15)	2.99(-0.30-6.39)	2.80(-0.65-6.38)
Argentina	1.12 (0.37-2.24)	26.37 (12.61-39.78)	1.67(-0.78-4.18)	1.75(-0.48-4.02)
Mongolia	1.12 (0.48-1.83)	26.19 (10.55-41.78)	1.61(0.29-2.96)	1.13(-0.03-2.30)
India	1.12 (0.45-1.91)	26.16 (9.64-43.91)	-0.10(-1.87-1.70)	0.27(-1.40-1.97)
Kyrgyzstan	1.12 (0.37-2.04)	26.15 (11.82-39.63)	3.85(0.23-7.60)	2.90(-0.50-6.41)
Iran (Islamic Republic of)	1.1 (0.56-1.7)	25.99 (13.02-39.7)	1.11(-0.14-2.37)	0.79(-0.34-1.94)
Comoros	1.09 (0.35-2.01)	25.93 (9.61-45.94)	1.33(-0.08-2.75)	1.35(-0.00-2.72)
Jordan	1.08 (0.46-1.76)	25.91 (13.27-39.02)	1.94(-1.11-5.08)	1.96(-0.86-4.87)
Benin	1.06 (0.38-1.8)	25.85 (10.3-41.13)	0.23(-0.66-1.12)	0.38(-0.44-1.20)
Guinea	1.04 (0.36-1.78)	25.8 (9.71-42.17)	1.87(-1.08-4.91)	1.41(-0.98-3.85)
Turkey	1.02 (0.34-1.79)	25.69 (13.41-38.96)	1.84(-1.13-4.90)	1.49(-1.18-4.24)
Eritrea	1.02 (0.36-1.8)	25.61 (8.53-47.58)	2.63(-1.66-7.10)	2.09(-1.79-6.13)
Lebanon	1.0 (0.3-1.83)	25.57 (12.24-38.67)	-0.59(-1.66-0.50)	-0.21(-1.24-0.83)
Yemen	0.97 (0.44-1.57)	24.37 (10.7-39.99)	2.24(-0.08-4.61)	2.06(-0.10-4.26)
Uganda	0.97 (0.45-1.49)	23.57 (7.56-41.45)	2.11(-2.39-6.81)	1.40(-2.26-5.21)
Chad	0.92 (0.24-1.87)	23.57 (8.64-39.99)	1.57(-1.38-4.61)	1.23(-1.49-4.02)
Republic of Korea	0.91 (0.2-1.88)	23.08 (8.27-40.97)	2.53(-1.18-6.37)	1.97(-1.45-5.51)
Afghanistan	0.88 (0.24-1.74)	22.89 (9.63-38.12)	1.73(-0.50-4.00)	1.37(-0.53-3.30)
Mali	0.87 (0.3-1.54)	22.37 (8.18-37.14)	0.88(-2.50-4.38)	0.90(-2.15-4.03)
Switzerland	0.84 (0.21-1.9)	22.1 (10.0-35.54)	0.90(-0.64-2.46)	0.55(-0.97-2.10)
Rwanda	0.84 (0.39-1.28)	21.95 (6.5-40.7)	0.95(-3.43-5.54)	1.15(-2.75-5.21)
South Sudan	0.79 (0.23-1.49)	21.83 (7.0-40.26)	3.28(-2.99-9.95)	2.32(-2.88-7.80)
Niger	0.75 (0.35-1.17)	21.72 (7.03-38.87)	0.79(-2.52-4.21)	0.82(-2.15-3.87)
Burundi	0.74 (0.22-1.45)	21.71 (6.83-41.25)	1.85(-2.95-6.88)	1.55(-2.70-5.98)
Uzbekistan	0.71 (0.33-1.12)	21.31 (9.17-34.51)	2.03(0.76-3.31)	1.36(0.15-2.58)
Papua New Guinea	0.71 (0.26-1.15)	21.19 (7.3-37.95)	1.22(-1.77-4.29)	1.28(-1.08-3.70)

Tajikistan	0.7 (0.3-1.14)	20.39 (8.78-32.77)	0.11(-1.22-1.46)	0.40(-0.90-1.72)
Japan	0.67 (0.16-1.49)	19.26 (7.21-31.87)	-0.90(-4.34-2.68)	-0.61(-3.87-2.77)
Somalia	0.55 (0.15-1.12)	18.88 (6.15-35.27)	1.66(-2.54-6.03)	1.61(-1.90-5.24)
Nepal	0.49 (0.23-0.73)	18.27 (5.74-34.04)	4.52(-0.73-10.06)	2.72(-1.46-7.07)
Ethiopia	0.43 (0.19-0.68)	16.74 (5.62-30.43)	2.38(-1.50-6.41)	2.61(-0.83-6.18)
Singapore	0.31 (0.13-0.52)	12.35 (4.97-21.06)	0.18(-3.06-3.53)	0.44(-0.85-1.75)

Table S4. Future trends in metabolic risks related to AF/AFL burden by sex.

Abbreviations: AF/AFL, atrial fibrillation /atrial flutter; ASDR, age-standardized rates of disability-adjusted life years; ASMR,age-standardized mortality rate.

Years	Female		Male		Both	
	ASDR(95%UI)	ASMR(95%UI)	ASDR(95%UI)	ASMR(95%UI)	ASDR(95%UI)	ASMR(95%UI)
1990	1.52(1.51-1.54)	1.52(1.51-1.54)	1.40(1.37-1.42)	1.40(1.37-1.42)	1.48(1.47-1.5)	1.48(1.47-1.5)
1991	1.53(1.52-1.55)	1.53(1.52-1.55)	1.40(1.38-1.42)	1.40(1.38-1.42)	1.49(1.48-1.5)	1.49(1.48-1.5)
1992	1.55(1.53-1.56)	1.55(1.53-1.56)	1.41(1.39-1.43)	1.41(1.39-1.43)	1.5(1.49-1.51)	1.50(1.49-1.51)
1993	1.57(1.55-1.58)	1.57(1.55-1.58)	1.42(1.4-1.44)	1.42(1.40-1.44)	1.52(1.51-1.53)	1.52(1.51-1.53)
1994	1.58(1.56-1.59)	1.58(1.56-1.59)	1.43(1.41-1.44)	1.43(1.41-1.44)	1.53(1.52-1.54)	1.53(1.52-1.54)
1995	1.59(1.58-1.60)	1.59(1.58-1.6)	1.44(1.42-1.45)	1.44(1.42-1.45)	1.54(1.53-1.55)	1.54(1.53-1.55)
1996	1.60(1.58-1.61)	1.60(1.58-1.61)	1.44(1.43-1.46)	1.44(1.43-1.46)	1.55(1.53-1.56)	1.55(1.53-1.56)
1997	1.60(1.58-1.61)	1.60(1.58-1.61)	1.45(1.43-1.46)	1.45(1.43-1.46)	1.55(1.53-1.56)	1.55(1.53-1.56)
1998	1.6(1.59-1.61)	1.60(1.59-1.61)	1.45(1.44-1.47)	1.45(1.44-1.47)	1.55(1.54-1.56)	1.55(1.54-1.56)
1999	1.61(1.59-1.62)	1.61(1.59-1.62)	1.46(1.44-1.47)	1.46(1.44-1.47)	1.56(1.55-1.57)	1.56(1.55-1.57)
2000	1.61(1.59-1.62)	1.61(1.59-1.62)	1.46(1.44-1.47)	1.46(1.44-1.47)	1.56(1.55-1.57)	1.56(1.55-1.57)
2001	1.61(1.59-1.62)	1.61(1.59-1.62)	1.46(1.44-1.47)	1.46(1.44-1.47)	1.56(1.55-1.57)	1.56(1.55-1.57)
2002	1.61(1.6-1.62)	1.61(1.60-1.62)	1.46(1.44-1.47)	1.46(1.44-1.47)	1.56(1.55-1.57)	1.56(1.55-1.57)
2003	1.61(1.6-1.62)	1.61(1.60-1.62)	1.46(1.45-1.48)	1.46(1.45-1.48)	1.56(1.55-1.57)	1.56(1.55-1.57)
2004	1.6(1.59-1.61)	1.6(1.59-1.61)	1.46(1.44-1.47)	1.46(1.44-1.47)	1.55(1.54-1.56)	1.55(1.54-1.56)
2005	1.59(1.58-1.61)	1.59(1.58-1.61)	1.46(1.44-1.47)	1.46(1.44-1.47)	1.55(1.54-1.56)	1.55(1.54-1.56)
2006	1.58(1.57-1.59)	1.58(1.57-1.59)	1.46(1.44-1.47)	1.46(1.44-1.47)	1.54(1.53-1.55)	1.54(1.53-1.55)
2007	1.58(1.57-1.59)	1.58(1.57-1.59)	1.46(1.45-1.48)	1.46(1.45-1.48)	1.54(1.53-1.55)	1.54(1.53-1.55)
2008	1.58(1.57-1.60)	1.58(1.57-1.6)	1.47(1.46-1.49)	1.47(1.46-1.49)	1.55(1.54-1.56)	1.55(1.54-1.56)
2009	1.58(1.57-1.60)	1.58(1.57-1.6)	1.48(1.47-1.49)	1.48(1.47-1.49)	1.55(1.54-1.56)	1.55(1.54-1.56)
2010	1.59(1.58-1.60)	1.59(1.58-1.6)	1.49(1.48-1.5)	1.49(1.48-1.50)	1.56(1.55-1.57)	1.56(1.55-1.57)

2011	1.59(1.57-1.60)	1.59(1.57-1.6)	1.49(1.48-1.51)	1.49(1.48-1.51)	1.56(1.55-1.56)	1.56(1.55-1.56)
2012	1.58(1.57-1.59)	1.58(1.57-1.59)	1.5(1.48-1.51)	1.5(1.48-1.51)	1.55(1.54-1.56)	1.55(1.54-1.56)
2013	1.58(1.57-1.59)	1.58(1.57-1.59)	1.5(1.49-1.52)	1.5(1.49-1.52)	1.56(1.55-1.57)	1.56(1.55-1.57)
2014	1.59(1.58-1.6)	1.59(1.58-1.6)	1.51(1.5-1.53)	1.51(1.50-1.53)	1.56(1.55-1.57)	1.56(1.55-1.57)
2015	1.59(1.58-1.6)	1.59(1.58-1.6)	1.52(1.5-1.53)	1.52(1.50-1.53)	1.57(1.56-1.58)	1.57(1.56-1.58)
2016	1.6(1.59-1.61)	1.6(1.59-1.61)	1.52(1.5-1.53)	1.52(1.50-1.53)	1.57(1.56-1.58)	1.57(1.56-1.58)
2017	1.6(1.59-1.61)	1.6(1.59-1.61)	1.52(1.51-1.53)	1.52(1.51-1.53)	1.58(1.57-1.59)	1.58(1.57-1.59)
2018	1.6(1.59-1.61)	1.6(1.59-1.61)	1.51(1.50-1.53)	1.51(1.50-1.53)	1.57(1.56-1.58)	1.57(1.56-1.58)
2019	1.6(1.58-1.61)	1.6(1.58-1.61)	1.51(1.50-1.52)	1.51(1.50-1.52)	1.57(1.56-1.58)	1.57(1.56-1.58)
2020	1.59(1.58-1.6)	1.59(1.58-1.6)	1.49(1.48-1.50)	1.49(1.48-1.5)	1.55(1.55-1.56)	1.55(1.55-1.56)
2021	1.57(1.56-1.58)	1.57(1.56-1.58)	1.48(1.46-1.49)	1.48(1.46-1.49)	1.54(1.53-1.55)	1.54(1.53-1.55)
2022	1.56(1.53-1.6)	1.56(1.53-1.6)	1.46(1.43-1.50)	1.46(1.43-1.5)	1.52(1.49-1.56)	1.52(1.49-1.56)
2023	1.55(1.5-1.6)	1.55(1.50-1.6)	1.45(1.40-1.50)	1.45(1.40-1.5)	1.51(1.46-1.56)	1.51(1.46-1.56)
2024	1.54(1.47-1.61)	1.54(1.47-1.61)	1.44(1.37-1.50)	1.44(1.37-1.5)	1.5(1.43-1.56)	1.5(1.43-1.56)
2025	1.53(1.44-1.63)	1.53(1.44-1.63)	1.42(1.33-1.51)	1.42(1.33-1.51)	1.49(1.40-1.57)	1.49(1.4-1.57)
2026	1.52(1.4-1.64)	1.52(1.40-1.64)	1.41(1.3-1.52)	1.41(1.30-1.52)	1.47(1.36-1.59)	1.47(1.36-1.59)
2027	1.51(1.36-1.66)	1.51(1.36-1.66)	1.4(1.26-1.53)	1.4(1.26-1.53)	1.46(1.33-1.6)	1.46(1.33-1.6)
2028	1.5(1.33-1.68)	1.5(1.33-1.68)	1.39(1.22-1.55)	1.39(1.22-1.55)	1.45(1.29-1.62)	1.45(1.29-1.62)
2029	1.49(1.29-1.7)	1.49(1.29-1.7)	1.37(1.18-1.57)	1.37(1.18-1.57)	1.44(1.25-1.63)	1.44(1.25-1.63)
2030	1.49(1.25-1.72)	1.49(1.25-1.72)	1.36(1.14-1.58)	1.36(1.14-1.58)	1.43(1.21-1.65)	1.43(1.21-1.65)

Figure S1. The burden of Metabolic Risk-Related AF/AFL by 21 GBD Regions by sex, in 1990 and 2021.

Abbreviations: AF/AFL, atrial fibrillation /atrial flutter; ASDR, age-standardized rates of disability-adjusted life years; ASMR,age-standardized mortality rate.

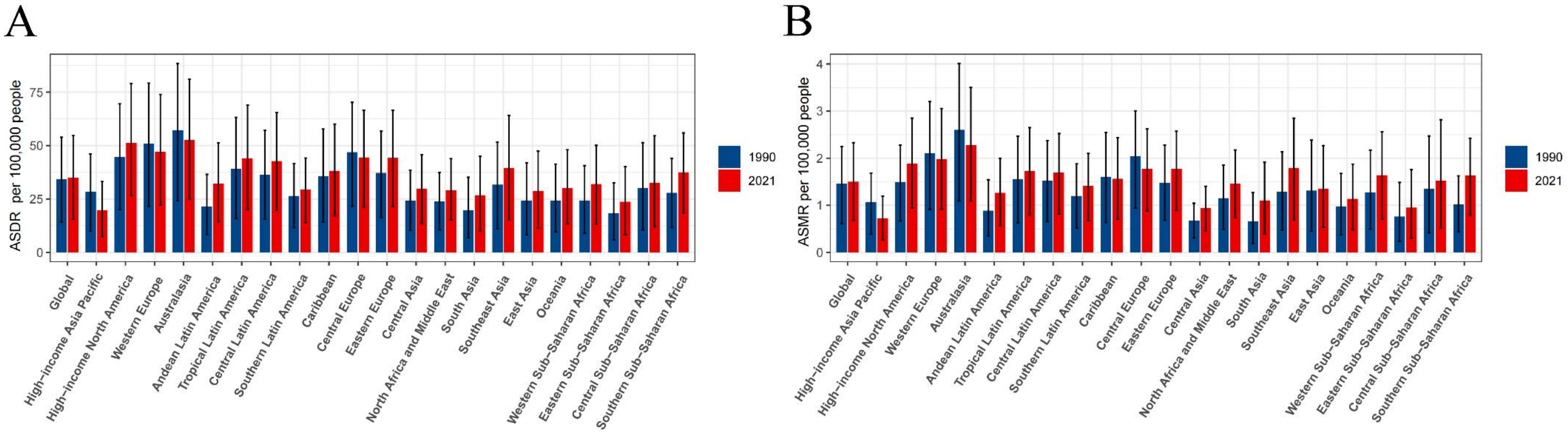

Figure S2. The burden of Metabolic Risk-Related AF/AFL by Country and SDI.

Abbreviations: AF/AFL, atrial fibrillation /atrial flutter; ASDR, age-standardized rates of disability-adjusted life years; ASMR,age-standardized mortality rate; SDI, socio-demographic index.Source: Global Burden of Disease Database, Institute for Health Metrics and Evaluation. The pathway was created using software in R.

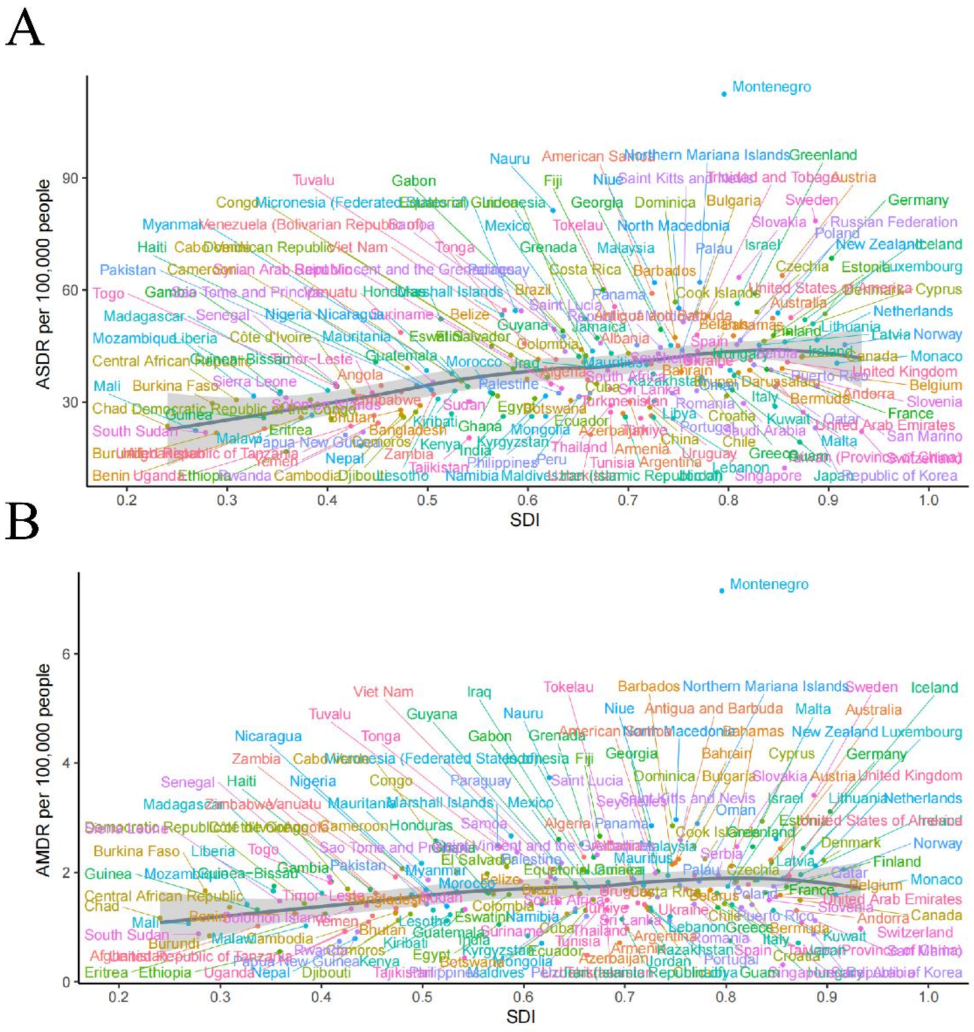

Supplement: Supplementary file 1 — Supplementary Material 1 [file 41598_2025_88744_MOESM1_ESM.pdf]
